# Inhibition of Proteasome LMP2 Activity Suppresses *Chil3* Expression in Mouse Colon Adenocarcinoma Tissue and Restrains Tumor Growth

**DOI:** 10.32604/or.2025.066611

**Published:** 2025-08-28

**Authors:** Tatiana M. Astakhova, Nikita S. Karpov, Nataliya O. Dashenkova, Elena V. Alpeeva, Mikhail V. Nesterchuk, Sergey B. Akopov, Arsen S. Mikaelyan, Anfisa S. Ryabchenko, Pavel A. Erokhov, Natalia P. Sharova

**Affiliations:** Koltzov Institute of Developmental Biology, Russian Academy of Sciences, Moscow, 119334, Russia

**Keywords:** Mouse colon 26 adenocarcinoma, M2 macrophages, proteasome low molecular mass protein 2 subunit, chitinase-3-like protein 3, KZR-504

## Abstract

**Objectives:**

Proteasomes, multi-subunit proteases, are key actors of cellular protein catabolism and a number of regulatory processes. The detection of subtle proteasome functioning in tumors may contribute to our understanding of the mechanisms of cancer development. The current study aimed to identify the role of low molecular mass protein 2 (LMP2), a proteasome immune subunit, in the development of mouse colon 26 (C26) adenocarcinoma.

**Methods:**

The functions of the LMP2 subunit in tumor development in Balb/c mice were studied using its irreversible inhibitor KZR-504. LMP2 activity was detected by the hydrolysis of the fluorogenic substrate Ac-Pro-Ala-Leu-AMC. Western blotting and Quantitative Reverse Transcription Polymerase Chain Reaction (qRT-PCR) were used. We applied fluorescent tests for cell proliferation and apoptosis. M2 macrophages were obtained by polarization of mouse bone marrow-derived macrophages using the corresponding cytokines.

**Results:**

KZR-504 showed high specificity only for the LMP2 subunit and had no negative effect on C26 cells in culture. However, KZR-504 suppressed the formation of tumor conglomerates (by 74%, *p* < 0.001) after C26 cell transplantation *in vivo*, inhibited the expression of chitinase-3-like protein 3 (Chil3) gene (by 90%, *p* < 0.001), a key marker of immunosuppressive M2 macrophages, in the tumor microenvironment, and reduced the tumor weight compared to the control (by 48%, *p* < 0.01). KZR-504 also suppressed the expression of *Chil3* (by 68%, *p* < 0.05) and arginase-1 (Arg1) (by 90%, *p* < 0.001), another marker gene, in M2 macrophages and violated M0-M2 macrophage polarization in culture.

**Conclusion:**

We discovered earlier unknown functions of the proteasome LMP2 subunit to facilitate the formation of tumor conglomerates and maintain *Chil3* and *Arg1* expression in immunosuppressive M2 macrophages. Our work demonstrates that the proteasome LMP2 subunit can be a target for antitumor treatment.

## 1 Introduction

Understanding the molecular mechanisms of tumor development largely depends on our knowledge of the intricacies of proteasome functioning. Proteasomes, multi-subunit proteases, are the main actors of intracellular protein catabolism. They are involved not only in the maintenance of proteostasis but also in the regulation of cellular processes. Nowadays, it is clear that the proteasome pool is changing in the development of malignant neoplasms, including thyroid cancer [1], squamous cell carcinoma [2], breast cancer [3], rectal adenocarcinoma [4]. To identify the general pattern of the proteasome functioning in cancer, it is necessary to conduct a detailed study of the proteasome pool in the specific types of tumors and clarify their significance not only for tumor cells but also for cells of the microenvironment.

Tumor tissues are enriched with macrophages [5]. The progression of some tumors is known to be associated with the switch of macrophage phenotype from M1 to M2 [6]. M1 macrophages perform an antitumor function, while M2 macrophages are involved in immune suppression, induction of hypoxia, promotion of angiogenesis, tumor cell proliferation, and metastasis [7]. M2 macrophage functions are closely related to the expression and secretion of chitinase-like proteins [8,9]. Specifically, chitinase-3-like protein 3 (Chil3), or Ym1, may play a role in macrophage activation at least through the fine-tuning of heparan sulfate levels in mice [8,10]. Therefore, the study of the regulation of Chil3 expression is of particular interest. Another important M2 macrophage marker is arginase-1 (Arg1) [8]. This enzyme produces L-ornithine and urea using the amino acid L-arginine as a substrate. By consuming L-arginine, Arg1 can suppress L-arginine-dependent functions of the immune system, such as T cell proliferation and, consequently, T cell response [8]. Both murine and human macrophages of the M2 type express Arg1. We believe that certain components of the tumor proteasome pool can be involved in the regulation of Chil3 and Arg1 expression.

In mammalian cells, the proteasome pool encompasses multiple proteasome forms that differ from each other in the structure and peculiarities of protein recognition and cleavage. Specifically, mammals possess constitutive (housekeeping), immune, mixed (intermediate), and some tissue-specific proteasomes [11]. Proteolytic subunits β1,β2, and β5 of the constitutive proteasomes exhibit caspase-like, trypsin-like, and chymotrypsin-like (ChTL) activities, respectively. The main function of the constitutive proteasomes is the maintenance of protein metabolism and the elimination of proteins with a damaged tertiary structure.

In the immune proteasomes, immune proteolytic subunits, namely, the low molecular mass protein 2 (LMP2) (β1i), the multicatalytic endopeptidase complex-like 1 (MECL1) (β2i), and LMP7 (β5i), are embedded instead of subunits β1, β2, and β5, respectively. The immune proteasomes display high trypsin-like and ChTL activities and low caspase-like activity. This is the reason for their producing several times more epitopes for the major histocompatibility complex (MHC) class I molecules than the constitutive ones.

The immune subunits can be integrated into proteasomes not only together with each other, but also in various combinations with constitutive subunits, producing mixed (or intermediate) forms [11–13]. Forms β1-β2-LMP7 (β5i) and LMP2 (β1i)-β2-LMP7 (β5i) were discovered in the human liver, colon, small intestine, kidney, tumor, and dendritic cells [12]. The existence of the mixed forms expands the variety of the antigens presented in the complex with MHC class I molecules to CD8 T cells [12].

The production of another mixed form, LMP2 (β1i)-MECL1 (β2i)-β5, is possible in LMP7-deficient mice under inflammatory conditions [13]. It is unclear whether this form exists in the cells under physiological conditions and we do not know what its role is.

In patients’ rectal cancer, the content of LMP2 and LMP7 proteasome subunits increases compared to the control tissue, and the rate of the increase in the content of LMP2 subunit significantly exceeds that of LMP7 subunit [4]. Moreover, expression levels of genes that encode proteasome beta subunits (*PSMB1-10*) were compared in normal tissue (n = 231), non-malignant colon adenomas (n = 132), and colorectal cancer (n = 342) with the use of the dataset E-MTAB-10089 [14]. Most of the *PSMB1-10* genes were revealed to be upregulated in adenomas and cancer compared to normal tissue; however, only the expression of *PSMB9*, encoding immune subunit LMP2 (β1i), was significantly higher in colorectal cancer compared to nonmalignant adenomas [15]. Based on all these data, we hypothesize that proteasomes containing the LMP2 subunit play a specific role in the development of gut cancer.

Proteasome structures differ not only in the set of proteolytic subunits but also in the presence of certain regulators (19S activator or 19S regulatory particle, proteasome activators PA28αβ/γ and PA200), which open the entry to the proteasome proteolytic chamber for definite substrates [11]. As a rule, the 19S activator recognizes ubiquitinated full-size protein substrates and promotes their entry into the proteolytic chamber. At the same time, other activators allow entry of intermediate-size and small polypeptides into the proteolytic chamber without preliminary ubiquitination. The damaged/oxidized proteins can overcome the hydrophobic barrier at the entrance to the proteolytic chamber of the 20S core without any activator [11].

The main goal of the current work was to investigate the role of the proteasome LMP2 subunit in the development of mouse colon 26 (C26) adenocarcinoma. It was also important to identify a proteasome activator that could be embedded in a common proteasome structure along with the LMP2 subunit. We paid special attention to immunological tolerance to tumor cells.

## 2 Materials and Methods

### 2.1 Animals

The study was carried out by EU Directive 2010/63/EU for animal experiments and granted ethical approval from the Ethics Committee for Animal Research of Koltzov Institute of Developmental Biology of the Russian Academy of Sciences (approval number: 63/20.10.2022). This ensures that the study adheres to the national and international guidelines for the care and use of laboratory animals. All methods were performed according to the relevant guidelines and regulations.

Balb/c mice (females of 1.5 months and 20–22 g) were purchased from the Stolbovaya Nursery (the Russian Academy of Sciences). The animals were acclimated to standard conditions for two weeks and kept in T-4\1 cages of 545 mm × 395 mm × 200 mm size (10 mice per cage) and T-2\2 cages of 318 mm × 202 mm × 135 mm size (up to 5 mice per cage) in Group of Biological Models of Koltzov Institute of Developmental Biology of the Russian Academy of Sciences. The Light mode was 12:12 (light from 8:00 a.m., dark from 8:00 p.m.) at a temperature of 23°C. We used “CHARA” foods for feeding laboratory rats and mice, and the animals received an unlimited amount of water. The cages were changed 1–2 times a week, depending on their contamination and the animals’ population size. All manipulations with mice were painless enough not to require any anesthesia. At the end of the experiment, mice were euthanized by CO_2_ inhalation in an automatic CO_2_ euthanizer-2M (AWTech, Moscow, Russia). CO_2_ concentration is 30% at the first stage and 70% at the second stage. The flow rate corresponds to the requirements of the American Veterinary Medical Association (AVMA) of 2020. The criteria established for euthanizing animals before the planned end of the experiment were tumors reaching 2 cm in size or animals developing diseases. We used 4–10 animals per experimental group. The total number of mice was 74.

### 2.2 Cells

C26 cells of murine colon adenocarcinoma were received from the Cell Culture Collection of Koltzov Institute of Developmental Biology of the Russian Academy of Sciences (Moscow, Russia). SW620 cells of human colon adenocarcinoma were received from the Engelhardt Institute of Molecular Biology of the Russian Academy of Sciences (Moscow, Russia). Cells were tested for mycoplasma contamination using Myco Real-Time Kit (#MR004, Evrogen, Moscow, Russia) in the Department of Cell Culture Collection of Koltzov Institute of Developmental Biology of the Russian Academy of Sciences. The tests showed no mycoplasma contamination. The authenticity (STR) of cells was confirmed by the Department of Cell Culture Collection of Koltzov Institute of Developmental Biology of the Russian Academy of Sciences. For all experiments, cells were used after the preliminary two passages.

Bone-marrow-derived macrophages were obtained from hematopoietic stem cells/progenitor cells of the bone marrow from the hind legs of Balb/c mice by an *in vitro* model for the study of macrophage polarization [16]. To induce differentiation of myeloid progenitors into mature macrophages, 10 ng/mL of macrophage colony-stimulating factor (M-CSF) (#130-094-129, Miltenyi Biotec, Inc., Bergisch Gladbach, Germany) was used. For M2 polarization, bone-marrow-derived macrophages (M0 macrophages) were incubated in a 20 ng/mL cocktail of interleukin 4 (IL-4) (#130-097-757, Miltenyi Biotec, Inc.) and IL-10 (#130-094-068, Miltenyi Biotec, Inc.) in complete growth media for 24 h. M2-polarized macrophages were used for investigations for a week. For M1 polarization, M0 macrophages were incubated in complete growth media containing 100 ng/mL lipopolysaccharide (LPS) (*E. coli*) (L2630, Sigma-Aldrich, St. Louis, MO, USA) for 3 and 27 h.

### 2.3 Treatment of Cells with KZR-504 and ONX-0914

C26 and SW620 cells were cultivated in Dulbecco’s Modified Eagle’s Medium (DMEM) containing glucose (4.5 g/L) (C420p, PanEco, Moscow, Russia), glutamine (GlutaMAX™, 100×; #35050061, Thermo Fisher Scientific, Waltham, MA, USA), 10% fetal bovine serum (FBS; FBS-11A, Capricorn Scientific, Ebsdorfergrund, Germany), at 37°C and 5% CO_2_ in the absence (control) and presence of 0.2 and 5 μM KZR-504 (HY-101786, MedChemExpress, Monmouth Junction, NJ, USA) for 24 and/or 48 h. Besides, C26 cells were cultivated in the same culture medium in the absence and presence of 50 μM KZR-504 and 5 μM ONX-0914 (HY-13207, MedChemExpress) for 24 and/or 48 h.

M2-polarized macrophages were cultivated in DMEM/F12 (C470p, PanEco) culture medium, containing 10% FBS, heat inactivated (A5670501, Thermo Fisher Scientific), and 20 ng/mL cocktail of IL-4 (#130-097-757, Miltenyi Biotec, Inc.) and IL-10 (#130-094-068, Miltenyi Biotec, Inc.), at 37°C and 5% CO_2_ in the absence (control) and presence of 5 μM KZR-504 for 48 h. In a number of experiments, a cocktail of IL-4 and IL-10 was introduced to a culture medium containing M0 macrophages, after the addition of 5 μM KZR-504. In experiments with M1 macrophages, 5 μM KZR-504 was introduced either simultaneously with LPS or after LPS addition.

### 2.4 Transplantation of Tumor Cells into Mice and Obtaining Samples of Tumor Tissue

C26 cells (10^6^) in 100 μL of physiological saline solution were subcutaneously transplanted (by injection) into the hip of female Balb/c mice (4 animals). The control was colon epithelial tissue of healthy females (4 animals). After three weeks, the developed tumors were extracted, weighed, and used for electrophoresis under non-denaturing conditions. Some of the tumor samples were stored at −70°C for two weeks. Subsequently, they were used for Western blotting.

To study the effect of KZR-504 on tumor conglomerate formation, mice were transplanted with C26 cells pre-incubated with 5 μM KZR-504 (model 1, 10 animals) and 50 μM KZR-504 (model 2, 10 animals) for 48 h. The control group with transplanted C26 cells in the absence of KZR-504 also contained 10 mice. To study the effect of KZR-504 on tumor development, the same three groups of animals were formed. Transplantation was performed as described above. KZR-504 (5 μM in 100 μL of physiological saline solution) was injected into the area of transplanted cells in model 1 daily for three weeks. The group of five mice (as an additional control) received an injection of 100 μL of physiological saline solution (instead of KZR-504 in 100 μL of physiological saline solution) into the tumor area. At the end of the experiment, the tumors were extracted, weighed, and used for Quantitative Reverse Transcription Polymerase Chain Reaction (qRT-PCR) and detection of LMP2 activity.

### 2.5 Preparation of Clarified Tissue Homogenates and Cell Lysates

Clarified homogenates of the colon epithelial tissue of healthy mice (control) and tumor tissues were prepared in six volumes (w/v) of buffer A containing 50 mM Tris-HCl (pH 7.5), 200 mM NaCl, 1 mM EDTA, 1 mM dithiothreitol, 10% glycerin, 5 mM MgCl_2_, 1 mM ATP, 10 mM Na_2_S_2_O_5_, leupeptin (0.5 μg/mL), pepstatin (1 μg/mL), and aprotinin (1 μg/mL) with the use of manual homogenizer with Teflon pestle 3213052 (#9.651854, Schuett-Biotec, Gottingen, Germany). The homogenates were centrifuged at 10,000 g for 30 min. All procedures were performed at 0°C–4°C. The supernatants (clarified homogenates) were used in the studies. Cell lysates were prepared in the same way, using 100 μL of buffer A per 5 × 10^6^ cells.

For electrophoresis under non-denaturing conditions, clarified tissue homogenates were prepared in three volumes (w/v) of buffer B containing 50 mM Na-HEPES, pH 7.5, 200 mM NaCl, and 10 mM EDTA as described earlier [17].

### 2.6 Detection of Proteasome Activities In Vitro

Proteasome LMP2 activity and ChTL activity were determined by the hydrolysis of the fluorogenic substrates Ac-Pro-Ala-Leu-AMC (Ac-PAL-AMC) (S-310, Boston Biochem, Cambridge, MA, USA) and N-succinyl-leu-leu-val-tyr-7-amido-4-methyl coumarin (Suc-LLVY-AMC) (S6510, Sigma-Aldrich), respectively. The reaction mixture contained 20 mM Tris-HCl (pH 7.5), 30 μM substrate, 1 mM dithiothreitol, 5 mM MgCl_2_, and 1 mM ATP. The reaction was carried out at 37°C for 20 min after adding 1.0, 2.0, 4.0, and 10 μL of clarified cell lysate or 1.0, 2.0, and 4.0 μL of clarified tissue homogenate or 0.5, 1.0 and 1.5 μL of purified human constitutive 20S proteasome (BML-PW7720, Enzo Life Sciences, New York, NY, USA) to a total volume of 100 μL and terminated by 1% SDS. To study the effect of the inhibitors, KZR-504 and ONX-0914 in different concentrations were pre-incubated with cell lysates or purified constitutive 20S proteasome at 37°C for 30 min before the start of the reaction. The product was detected by using a VersaFluor^TM^ Fluorometer (#170-2402, Bio-Rad Laboratories, Inc., Hercules, CA, USA) with an excitation wavelength of 380 nm and an emission wavelength of 440 nm. The activity was calculated for 10 μL of clarified lysate, 1 μL of clarified tissue homogenate or 1 μL of purified constitutive 20S proteasome. Under the indicated conditions, the quantity of the reaction product is proportional to the reaction duration [17].

### 2.7 Detection of LMP2 Activity in Non-Denaturing Gel

LMP2 activity in non-denaturing gradient 4–10% polyacrylamide gel (5 μL of clarified homogenate or 60–70 μg of protein per lane) was detected with the use of fluorogenic 300 μM Ac-PAL-AMC in 200 mM Na-HEPES buffer, pH 7.5 (1/20 of gel volume) after electrophoresis at 60 V for 14 h, 140 V for 10 h, and 260 V for 20 h, as described previously [17]. Thyroglobulin (670 kDa), labeled with dye Cy-3.5, was used as a marker of the molecular mass. Fluorescence bands in the gel were photographed (manual exposure, photo camera Nikon D5200, Nikon, Sendai, Japan) under UV light with a wavelength of 365 nm. The image analysis of LMP2 activity in the gel was performed using the standard ImageJ software, v.1.54g (an open-source software developed by contributors worldwide, which is available at any time in the public domain and distributed under the BSD-2 license https://imagej.net/Welcome, accessed on 12 July 2025).

### 2.8 Western Blot Analysis

For Western blotting, clarified tissue homogenates and cell lysates were prepared as described in Section 2.5. The protein concentration was determined by the standard Lowry method. After SDS electrophoresis of clarified tissue homogenates (10 μL or 114–132 μg of protein per lane) or C26 cell lysates (5–20 μL or 18–72 μg of protein per lane) in 13% or 8% polyacrylamide gel, polypeptides were transferred from the gel onto nitrocellulose membrane (#1620115, Bio-Rad Laboratories, Inc.) by the standard procedure. Immunodetection was carried out with the use of primary combined mouse monoclonal antibodies (mAb) to proteasome α1, 2, 3, 5, 6 & 7 subunits (BML-PW8195), mouse mAb to LMP7 subunit (BML-PW8845), mouse mAb to LMP2 subunit (BML-PW8840), mouse mAb to proteasome 19S regulatory particle ATPase subunit 6 (Rpt6) (BML-PW9265), rabbit polyclonal antibodies (pAb) to proteasome 11S subunit PA28α (BML-PW8185) (all Enzo Life Sciences; all 1:1500), rabbit pAb Anti-PSMB5 (β5 subunit; GTX23330, 1:2000, GeneTex, Irvine, CA, USA), rabbit mAb Anti-PSMB6 (β1 subunit; #13267, 1:1000, Cell Signaling, Danvers, MA, USA), rabbit Anti-CD163 Ab (ab182422, 1:1000, Abcam Limited, Cambridge, UK), mouse mAb to β-actin (ab8226, 1:1000, Abcam Limited), and corresponding secondary Ab peroxidase conjugated (ECL Western Blotting Detection Kit) (RPN 2108, 1:2000, Danaher Corp., Amersham, UK). The nitrocellulose membrane was treated with the primary and secondary Ab for 1.5 h and 1 h, respectively, at a temperature of 23°C.

SDS electrophoresis of macrophage lysates was performed in 8% polyacrylamide gel (10 μL or 25–27 μg of protein per lane). For immunodetection of polypeptides on the nitrocellulose membrane, rabbit pAb to Chil3 (PA5-81356, 1:2000, Thermo Fisher Scientific) were used. The image analysis was performed using ImageJ software.

### 2.9 Assay for Cytotoxic Effect

The cytotoxic effect of KZR-504 on C26 and SW620 cells was evaluated with the use of the standard colorimetric assay based on the ability of viable cells to transform 3-(4,5-dimethylthiazol-2yl)-2, 5-diphenyltetrazolium bromide (MTT) to purple formazan crystals soluble in organic solvents. After incubating cells (5000 cells per probe) in culture media in the absence or presence of 0.2 and 5 μM KZR-504 or 5 μM ONX-0914 for 24 h/48 h, 0.45 mg/mL MTT was added to the probes and kept in a CO_2_ incubator for 4 h at 37°C. The culture medium was replaced with 0.5% dimethyl sulfoxide for 1 h at room temperature. The quantity of formazan (proportional to the number of viable cells) was measured as absorbance at 550 nm. The reference wavelength was set at 620 nm. The final calculation was the subtraction of the absorbance magnitude at 620 nm from that at 550 nm. The number of viable cells in the presence of KZR-504 or ONX-0914 was presented as a percentage of the control magnitude (without effectors).

### 2.10 Test for Cell Proliferation

Cells that underwent mitosis were detected with the use of bromodeoxyuridine (BrdU) and 4^′^,6-diamidino-2-phenylindole (DAPI). BrdU is embedded in the newly synthesized DNA chain instead of thymidine. DAPI, a blue fluorescent dye, binds strongly to adenine and thymine-rich regions of DNA. DAPI is convenient to use for staining fixed cells. Co-localization of BrdU and DAPI indicates cell proliferative activity.

C26 Cells (20,000 cells per sample) were incubated in the presence of BrdU (10 μM) for 1 h, washed with 0.02 M phosphate-buffered saline (PBS), pH 7.0, fixed with 4% paraformaldehyde for 5 min, and washed again. To bind to DAPI, the DNA of fixed cells was preliminarily hydrolyzed by treatment with 2.5 M HCl for 1 h. Samples washed in PBS were treated with primary rat Ab against BrdU (ab6326, 1:250, Abcam Limited) for 18 h at 4°C in a humid chamber. After washing, cells were incubated with secondary Ab, Alexa Fluor 546 goat anti-rat IgG (A-11081, 1:1000, Thermo Fisher Scientific) for 1.5 h in the absence of light, washed with PBS and incubated with DAPI (#40011, Biotium, Fremont, CA, USA) (5 μM) for 10 min. After fluorescence microscopy (Olympus IX73 microscope with DP74 camera; Olympus Corporation, Hachioji-shi, Japan) visualization, cell images were analyzed with the use of ImageJ software and the CellCounter tool, v.2.

### 2.11 Test for Cell Apoptosis

The fluorogenic substrate CellEvent™ Caspase 3/7 Green (R37111, Thermo Fisher Scientific) was used to detect cells in the state of apoptosis. This substrate is processed by active caspase 3/7, and reaction products fluoresce green. C26 cells (20,000 cells per sample) were incubated in a complete growth medium with substrate CellEvent™ Caspase 3/7 Green (2 drops of substrate per 1 mL of medium) for 30 min. After washing the cells with 0.02 M PBS, pH 7.1, 5 μg/mL of Hoechst 33342 (Thermo Fisher Scientific) was added to the cultivation medium for 15 min. Hoechst 33342 is a dye that stains the nuclei of living cells and fluoresces blue. After fluorescence microscopy visualization, cell images were analyzed with the use of ImageJ software and the CellCounter tool.

### 2.12 qRT-PCR

Total RNA was isolated from mouse tumors and macrophages using TRI-Reagent (Sigma-Aldrich) by the manufacturer’s protocol. The concentration and quality of the isolated total RNA were determined using a NanoDrop 2000 spectrophotometer (Thermo Fisher Scientific) at a wavelength of 260 nm. The RNA quality was confirmed by electrophoresis on a GelDoc XR analyzer (Bio-Rad Laboratories, Inc.). For cDNA synthesis, 1 μg of total RNA, oligoDT primers, and reagents RevertAid RT Kit (Thermo Fisher Scientific) were used according to the manufacturer’s protocol. qRT-PCR was performed on Applied Biosystems StepOne Plus Real-Time PCR System (Thermo Fisher Scientific) using qPCRmix-HS SYBR master mix containing SYBR Green I dye (Evrogen, Moscow, Russia). The primers used are shown in Table 1.

**Table 1:** List of mouse primers used for qRT-PCR

Gene	GenBank acces-	Primer sequence	
		name	sion number
Forward, 5^′^→3^′^	Reverse, 5^′^→3^′^		
Gapdh	NM_001289726	GCCCATCACCATCTTCCA	TTCACACCCATCACAAACAT
Eef2	NM_007907.3	GCCTATCCAGAGAACCAT	CGTCTTCACAAGGAACTG
Chil3	NM_009892.4	GCTCAGTGTTCTTGTCTT	GCTGATGTGCTACTATACC
Arg1	NM_007482.3	AATTTACAAGACAGGGCTCC	GCATTCACAGTCACTTAGGT
IL-6	NM_031168.2	GACTGATGCTGGTGACAA	GCCATTGCACAACTCTTT

Note: Gapdh, Glyceraldehyde 3-phosphate dehydrogenase; Eef2, Eukaryotic elongation factor 2; Chil3, Chitinase-3-like protein 3; Arg1, Arginase-1; IL-6, Interleukin 6.

Primers were designed and tested using the software Beacon Designer, v.8.21 (Premier Biosoft, San Francisco, CA, USA), DNAstar, v.11.2.1 (Madison, WI, USA), open international databases NCBI (https://www.ncbi.nlm.nih.gov/, Bethesda, MD, USA), and UCSC (https://genome.ucsc.edu/, Santa Cruz, CA, USA). The annealing temperature was 60°C. The data on the expression of the studied genes were normalized to the expression of Gapdh and Eef2 housekeeping genes.

### 2.13 Immunohistochemistry

Tumor samples were isolated and immersed in 4% (w/v) paraformaldehyde (PAF; #211511, PanReac AppliChem, Darmstadt, Germany) solution in 0.02 M PBS (B-60251, Eco Service, Saint-Petersburg, Russia), pH 7.4, for 2 h at 23°C for fixation. After that, the samples were immersed in a 25% (w/v) sucrose (#84097, Sigma-Aldrich) solution in 0.02 M PBS for 24 h for cryoprotection and then frozen in hexane cooled to −40°C with liquid nitrogen. Non-serial 10 μm-thick sections were prepared using a cryostat-microtome Leica CM1900 (Leica Microsystems, Wetzlar, Germany) at −23–25°C, mounted on Superfrost Plus slides (#22-037-246, Thermo Fisher Scientific), and dried at 23°C. The sections were rinsed in 0.01 M PBS with 0.3% Triton X-100 (PBST; X100-5ML, Sigma-Aldrich) and incubated in a wet chamber sequentially with: (1) PBST containing 1% (w/v) bovine serum albumin (BSA; #9048-46-8, Sigma-Aldrich), (2) primary rabbit Anti-CD163 Ab (ab182422, 1:500, Abcam Limited) in PBST with 1% BSA for 48 h at 4°C, and (3) secondary goat antirabbit IgG conjugated with AlexaFluor 488 (ab150077, 1:500, Abcam Limited) in PBST with 1% BSA (pH 7.2–7.4) for 2 h at 20°C. The sections were rinsed three times for 10 min in PBST after the primary Ab and in PBS after the secondary Ab. After being rinsed, the samples were mounted in Mowiol (#81381, Sigma-Aldrich). The sections were visualized with a confocal microscope Leica TCS SP5 (Leica Microsystems, Wetzlar, Germany) using an excitation wavelength of 488 and a differential interference contrast (DIC) system.

### 2.14 Statistics

The statistical analysis was carried out in GraphPad Prism 9.0 (GraphPad Software, Inc., San Diego, CA, USA) with the use of ordinary one-way ANOVA. All data were expressed as mean ± standard deviation. Differences between groups were detected by Student’s *t*-test. Significant difference was considered at *p* < 0.05 (95% confidence interval).

## 3 Results

### 3.1 Patterns of the Expression of Proteasome Subunits in Mouse Colon 26 Adenocarcinoma

First of all, we tested the validity of using mouse C26 cells for the creation of models of gut cancer development. We investigated changes in the content of the LMP2 subunit in comparison with that of the LMP7 subunit in the tumor developed after subcutaneous transplantation of C26 cells in Balb/c mice. In addition to LMP2 and LMP7 immune subunits, we investigated the relative content of the total proteasome pool and proteasome activators PA28αβ and 19S in the samples of the colon 26 adenocarcinoma tissue in comparison with that of the colon epithelial tissue of healthy Balb/c mice.

**Figure 1:**
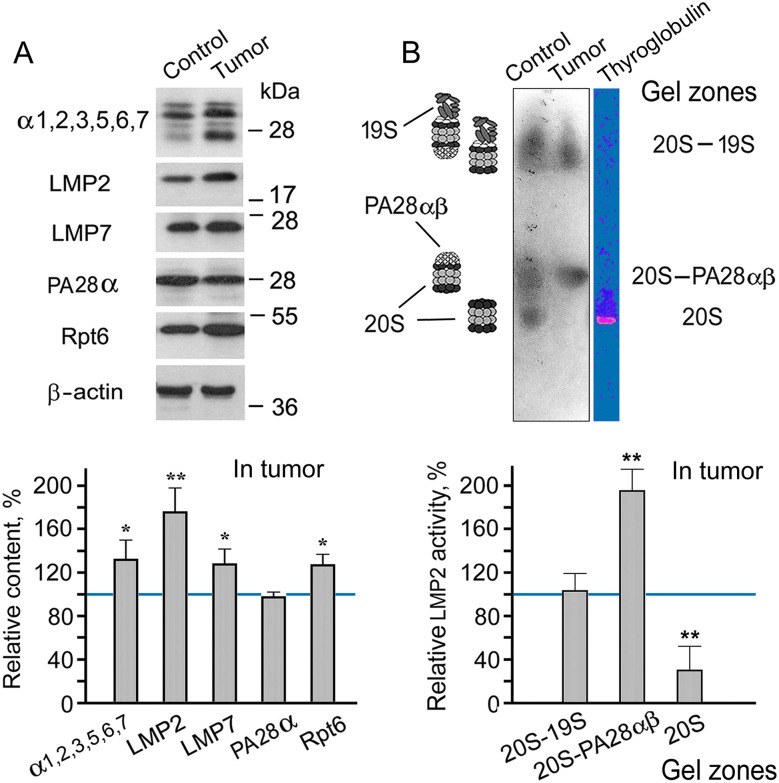
Patterns of proteasome subunit content and low molecular mass protein 2 (LMP2) activity in colon adenocarcinoma developed after subcutaneous transplantation of C26 cells into mice. (**A**) Western blots and relative content of proteasome subunits in cleared homogenates normalized to β-actin level. The molecular mass of the standard protein markers is shown. The blots were cropped horizontally from gels according to the molecular mass of the proteins studied. Original full-length gels are shown in Fig. S1. (**B**) LMP2 activity in the non-denaturing gel. The image of the non-denaturing gel was cropped horizontally to obtain the fragment containing zones of LMP2 activity. The original full-length gel is shown in Fig. S3. The subunit content and LMP2 activity in cleared homogenates of the control colon epithelial tissue of healthy mice were taken as 100%. The standard deviation is shown. Significant difference from the corresponding control at *p* < 0.05 (*) and *p* < 0.01 (**), n = 4. 20S, proteasome core particle; 19S, proteasome activator (regulatory particle) 19S; PA28αβ, proteasome activator PA28αβ; α1, 2, 3, 5, 6, 7, subunits of proteasome α rings; Rpt6, 19S regulatory particle ATPase subunit 6; PA28α, subunit of PA28αβ activator

The content of the total proteasome pool was assessed by the level of α1, 2, 3, 5, 6, 7 subunits, which are present in all proteasome forms. The amount of PA28αβ and 19S activators was evaluated by the level of their subunits, PA28α and Rpt6, respectively. The content of the total proteasome pool, immune subunits, and 19S activator was revealed to increase in the adenocarcinoma (Figs. 1A, S1 and S2, Table S1). The additional amount of 19S activator at least provides elevated ubiquitin-dependent protein catabolism in the tumor. The increased total proteasome pool reflects the high content of proteasomes containing LMP2 and/or LMP7 immune subunits.

It is very important that the rate of increase in the amount of LMP2 subunit in the tumor is greater than that of the LMP7 subunit. This fact confirms the validity of using mouse C26 cells for the creation of models of gut cancer development. Previously, we had obtained a similar result for patients with rectal cancer [4]. This result suggests the existence of a proteasome form containing LMP2 (without LMP7) subunit in addition to other proteasome forms in tumor tissue. It is likely to be the form that had been identified in LMP7-deficient mice under inflammatory conditions [13].

Which of the proteasome activators is associated with the increased content of the proteasomes involving the LMP2 subunit? To answer this question, we investigated the activity of the LMP2 subunit in a gel after electrophoresis of cleared tissue homogenates in non-denaturing conditions [17]. The proteasomes exhibiting LMP2 activity in cleared homogenates of mouse control and tumor tissues were localized in three gel zones (Fig. 1B). The proteasomes containing the 19S activator had the least mobility in the gel. The proteasomes that did not contain any activator had the greatest mobility. The proteasomes containing the PA28αβ activator, without the 19S activator, showed intermediate mobility [17]. It is of great interest that LMP2 activity was enhanced in the proteasome fraction containing PA28αβ activator (Figs. 1B, S2 and S3, Table S2). Since the expression of the PA28αβ activator did not enhance in the tumor (Fig. 1A), a redistribution of this activator probably occurred via increasing its binding to the proteasome structures with the LMP2 subunit. It is noteworthy that the most significant increase in LMP2 activity was associated with the PA28αβ activator, also in the patients’ rectal cancer [4].

On the whole, the results obtained in the present work provide a basis to investigate proteasome LMP2 subunit functions in mouse colon adenocarcinoma with the use of LMP2 inhibitor, and allow us to link the findings to proteasome mechanisms of gut cancer development in patients.

### 3.2 Choice of KZR-504 Concentrations for Experiments in Cellulo and In Vivo

**Figure 2:**
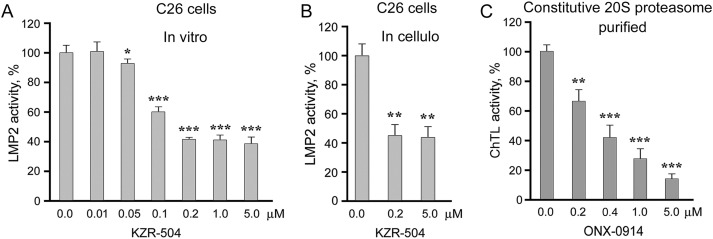
Effects of proteasome inhibitors on proteasome activities. (**A**) Effect of KZR-504 on LMP2 activity in clarified lysates of C26 cells. (**B**) Effect of KZR-504 on LMP2 activity in C26 cells after 24 h cultivation. (**C**) Effect of ONX-0914 on ChTL activity of purified constitutive 20S proteasome. The standard deviation is shown. Significant difference from the corresponding control at *p* < 0.05 (*), *p* < 0.01 (**), and *p* < 0.001 (***); for (**A**) and (**C**), n = 5, and for (**B**), n = 4

To select concentrations of KZR-504, an irreversible inhibitor of LMP2 subunit, for experiments *in cellulo* and *in vivo*, we determined its concentrations that effectively suppressed LMP2 activity in clarified lysates of C26 cells (*in vitro*) (Fig. 2A). KZR-504 maximally suppressed LMP2 activity starting from a concentration of 0.2 μM. At higher concentrations, the effect reached a plateau.

We chose not only low (0.2 μM) but also higher (5 μM) concentrations of KZR-504 to study its effects *in cellulo* and *in vivo*. The concentration of 5 μM is needed to create an *in vivo* mouse model in which the activity of the LMP2 subunit is reliably inhibited to the maximal degree, and this concentration is not toxic for the mouse organism. *In vivo*, the inhibitor injected in the tumor site spreads over the body, and thus its concentration in the tumor site decreases. Therefore, we chose the excess concentration of 5 μM. *In cellulo*, KZR-504 at both concentrations suppressed LMP2 activity to the same extent as *in vitro* (Fig. 2B), indicating good permeability of the cell membrane for this inhibitor.

For positive control, we chose 5 μM ONX-0914 that strongly inhibited ChTL activity of the constitutive β5 subunit (Fig. 2C), important for ensuring the viability of different types of malignant cells [18–20].

### 3.3 Effect of KZR-504 on C26 and SW620 Cells

**Figure 3:**
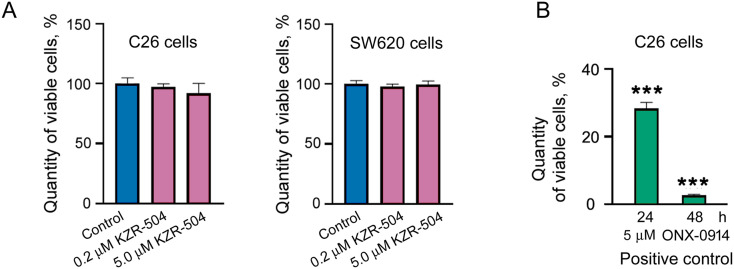

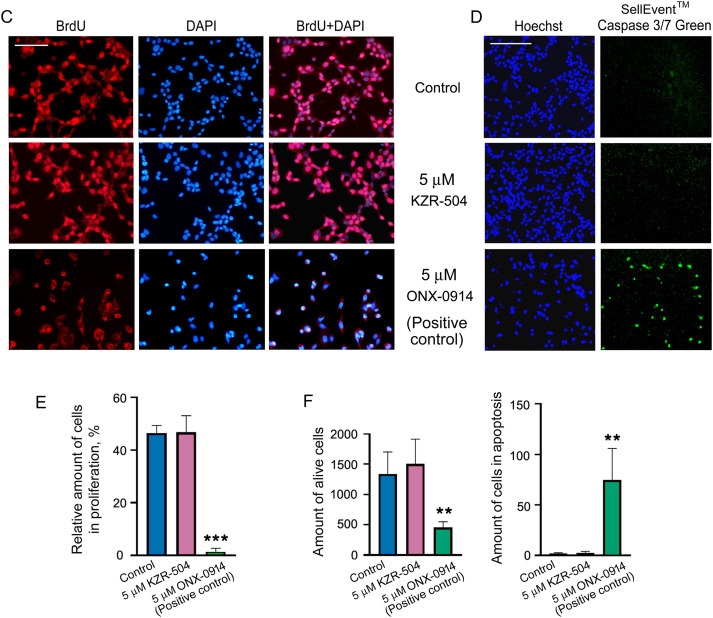
Effect of KZR-504 on C26 and SW620 cells. (**A**) The number of viable C26 and SW620 cells after their 24 h cultivation in the absence (control) and presence of different concentrations of KZR-504 (3-(4,5-dimethylthiazol-2yl)-2,5-diphenyltetrazolium bromide (MTT) test). (**B**) The number of viable C26 cells after their 24 h and 48 h cultivation in the presence of 5 μM ONX-0914 (the positive control for MTT test). (**C**) Images of C26 cells labeled with 4^′^,6-diamidino-2-phenylindole (DAPI) and antibodies against bromodeoxyuridine (BrdU) (proliferation test) after their 24 h cultivation in the absence (control) and presence of 5 μM KZR-504 and 5 μM ONX-0914 (the positive control). (**D**) Images of C26 cells labeled with Hoechst 33342 (alive cell test) and CellEvent™ Caspase 3/7 Green (apoptosis test) after their 24 h cultivation in the absence (control) and presence of 5 μM KZR-504 and 5 μM ONX-0914 (the positive control). Scale bar, 100 μm. Original microscopy images of cells are shown in Figs. S4 and S5. (**E**) The relative amount of cells involved in proliferation (the number of cells labeled with antibodies against BrdU relative to the number of cells labeled with DAPI). (**F**) The number of alive cells and cells involved in apoptosis. The standard deviation is shown. Significant difference with control at *p* < 0.01 (**) and *p* < 0.001 (***); for (**A**) and (**B**), n = 10; for (**E**), n = 4; and for (**F**), n = 6

KZR-504 at the concentration of 0.2 μM, as well as at the concentration of 5 μM, did not affect C26 cell viability (Fig. 3A) in contrast to 5 μM ONX-0914 taken as a positive control (Figs. 2C and 3B). We also tested the effect of KZR-504 at both concentrations on SW620 cells of human colon cancer. The viability of SW620 cells did not change under the influence of this inhibitor (Fig. 3A). Obviously, the viability of colon cancer cells of mice and humans in the culture does not depend on the proteasome LMP2 subunit.

To make sure that KZR-504 does not act on C26 cells, we tested its ability to affect cellular proliferation and apoptosis. The number of proliferating cells in the culture was assessed by fluorescence microscopy visualization. Cells, that passed mitotic divisions, had nuclei colored in red (anti-BrdU-Ab staining) and blue (DAPI staining), and cells that did not divide had nuclei colored only in blue (Figs. 3C and S4). The total number of cells in the culture and the number of cells undergoing apoptosis were also evaluated by fluorescence microscopy visualization (Figs. 3D and S5). KZR-504 had no effect on C26 cell proliferation and apoptosis, and did not reduce the cell amount (Fig. 3C–F). In contrast, 5 μM ONX-0914, taken as a positive control, dramatically suppressed C26 cell proliferation, induced apoptosis, and reduced the cell number in the culture (Fig. 3C–F). Thus, the proteasome LMP2 subunit of colon adenocarcinoma cells is not involved in maintaining their viability or proliferative activity in the culture (*in cellulo*).

### 3.4 Creation of In Vivo Models of Colon 26 Adenocarcinoma Development under Conditions of Altered LMP2 Activity

The high expression of the LMP2 subunit in the colon adenocarcinoma tissue (Fig. 1A) may be explained by its significance for tumor cells not *in cellulo*, but *in vivo*, being implemented through the microenvironment. If this is the case, there are three possibilities for the proteasome LMP2 subunit to participate in the colon adenocarcinoma development. First, the proteasome LMP2 subunit localized in C26 cells may be important for this process. Second, the proteasome LMP2 subunit of microenvironment cells may be involved in tumor development. Third, both assumptions may be true. To check these possibilities, we created *in vivo* models of colon adenocarcinoma development under conditions of altered LMP2 activity in the microenvironment and/or tumor cells.

**Figure 4:**
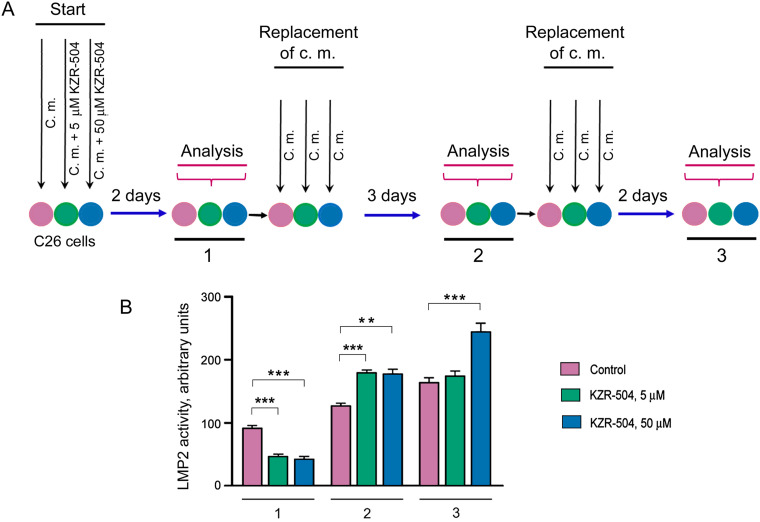

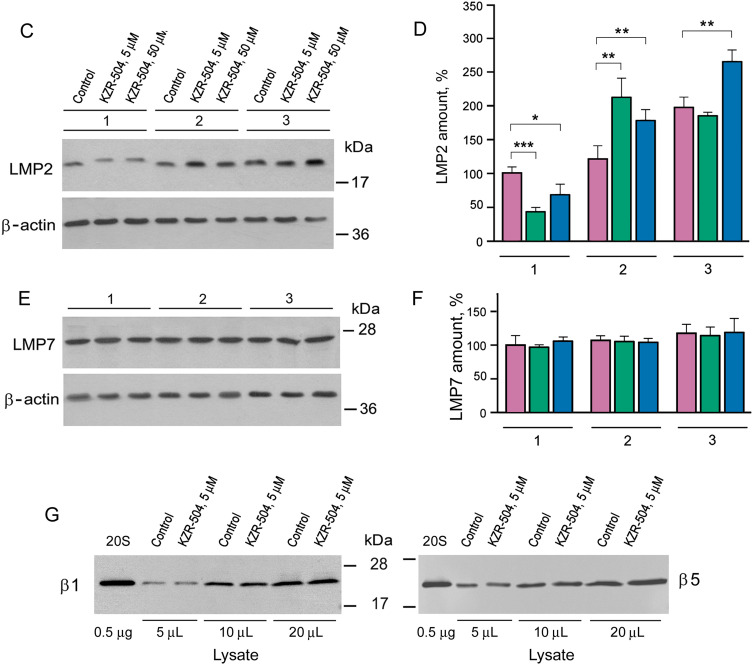
Determination of the duration of KZR-504’s effect on proteasome subunits in C26 cells. (**A**) The schematics of the experiment. C. m., culture medium. 1, 2, 3—the first, second, and third stages of analysis. Stage 1: Two days after the start of the cultivation of C26 cells in the absence (control) and presence of 5 μM and 50 μM KZR-504. Stage 2: Three days after the exclusion of KZR-504 from the culture medium. Stage 3: Five days after the exclusion of KZR-504 from the culture medium. (**B**) Activity of the LMP2 subunit. (**C**) Western blots of the LMP2 subunit. (**D**) Relative amount of LMP2 subunit. (**E**) Western blots of the LMP7 subunit. (**F**) Relative amount of LMP7 subunit. (**G**) Western blots of β1 and β5 subunits after two days of the cultivation of C26 cells in the absence (control) and presence of 5 μM KZR-504. 20S, purified constitutive 20S proteasome, 0.5 μg. The molecular mass of the standard protein markers is shown. All the blots were cropped horizontally from gels according to the molecular mass of the proteins studied. Original full-length gels are shown in Fig. S6. The number of subunits in C26 cells after their cultivation during the first two days in the absence of KZR-504 was taken as 100%. The standard deviation is shown. Significant difference at *p* < 0.05 (*), *p* < 0.01 (**), and *p* < 0.001 (***), n = 4

Initially, we analyzed the duration of the KZR-504 effect on the LMP2 subunit in C26 cells in accordance with the schematics shown in Fig. 4A. In addition to 5 μM KZR-504, we tested the effect of 50 μM KZR-504 to reveal a set of KZR-504 concentrations for *in vivo* models. The scheme included three stages of analysis of LMP2 activity and amount. At the first stage, LMP2 activity and amount were evaluated two days after the start of the cultivation of C26 cells in the absence (control) and presence of 5 and 50 μM KZR-504. After that, the culture medium was replaced with a new portion in the absence of KZR-504 in all samples. Three days later, LMP2 activity and amount were analyzed (the second stage). The culture medium was replaced again, and two days later, the analysis was repeated (the third stage). Thus, LMP2 activity and content were evaluated three days and five days after the inhibitor was excluded from the culture medium. These periods were important, as the tumor conglomerate had formed between the third and fifth days after subcutaneous transplantation of C26 cells into mice.

At stage 1, LMP2 activity was detected to decrease by 50% and 56% respectively in the presence of 5 and 50 μM KZR-504 in the culture medium (Fig. 4B). At stage 2, three days after the exclusion of 5 and 50 μM KZR-504 from the culture medium, LMP2 activity was enhanced to the same level in both samples. At stage 3, five days after the exclusion of 5 μM KZR-504 from the culture medium, LMP2 activity did not differ from the control value. However, in the sample of the initial 50 μM KZR-504 that was removed afterward, it remained increased.

It should be underscored that the irreversible binding of KZR-504 to the LMP2 subunit slowed down its mobility in the gel (Figs. 4C, S6 and S7). This fact is well known for other specific irreversible inhibitors of the LMP2 subunit [21]. The amount of such abnormal subunits decreased (Figs. 4C,D and S7, Table S3). Three days later, abnormal LMP2 subunits were not revealed (Figs. 4C and S7). Instead, an excessive amount of normal LMP2 subunit was expressed. Two days thereafter, the LMP2 amount decreased to the control level (stage 3; initial 5 μM KZR-504) or remained high (stage 3; initial 50 μM KZR-504). These changes coincided with changes in LMP2 activity. Importantly, KZR-504 did not change LMP7 mobility in the gel and did not influence LMP7 content (Figs. 4E,F and S7, Table S3). KZR-504 did not change the electrophoretic mobility of β1 and β5 constitutive subunits in C26 cells (Fig. 4G). For this experiment, a purified constitutive 20S proteasome was used as a standard. Thus, KZR-504 showed high specificity concerning the LMP2 subunit in our work.

The dynamics of change in the content of LMP2 and LMP7 subunits differed greatly (Fig. 4D,F, Table S3). This indicates the presence of proteasome structures containing the LMP2 subunit without the LMP7 subunit in C26 cells.

Based on the results obtained, we have created two *in vivo* models of the colon adenocarcinoma development under conditions of altered LMP2 activity (Table 2). In model 1, the activity of the LMP2 subunit was inhibited at the formation of tumor conglomerates and during tumor development in mice within three weeks after subcutaneous transplantation of C26 cells. Preliminary, C26 cells were pre-incubated with 5 μM KZR-504 for 48 h. During tumor development, KZR-504 (5 μM) was injected into the area of transplanted C26 cells on a daily basis. In model 2, the LMP2 subunit was activated, at least during the formation of tumor conglomerates. For this model, C26 cells were pre-incubated with 50 μM KZR-504 for 48 h. After subcutaneous transplantation of C26 cells to mice, KZR-504 was not injected.

**Table 2:** *In vivo* models of the development of C26 adenocarcinomas under conditions of altered LMP2 activity

Model	Description of manipulations		
	Pre-Incubation of C26 Cells, 48 h	Subcutaneous transplantation of 10^6^ C26 cells to mice	Daily treatment
Control	−KZR-504	−KZR-504	−KZR-504
Model 1	+KZR-504, 5 μM	+KZR-504, 5 μM	+KZR-504, 5 μM
Model 2	+KZR-504, 50 μM	−KZR-504	−KZR-504

Thus, model 1 was useful for testing the possibility of the involvement of the proteasome LMP2 subunit localized in microenvironment cells in the tumor development. Model 2 allowed us to test the possibility of the participation of the proteasome LMP2 subunit localized in C26 cells in the formation of tumor conglomerates.

### 3.5 Features of the Formation and Development of Tumor Conglomerates in Model 1 and Model 2

**Figure 5:**
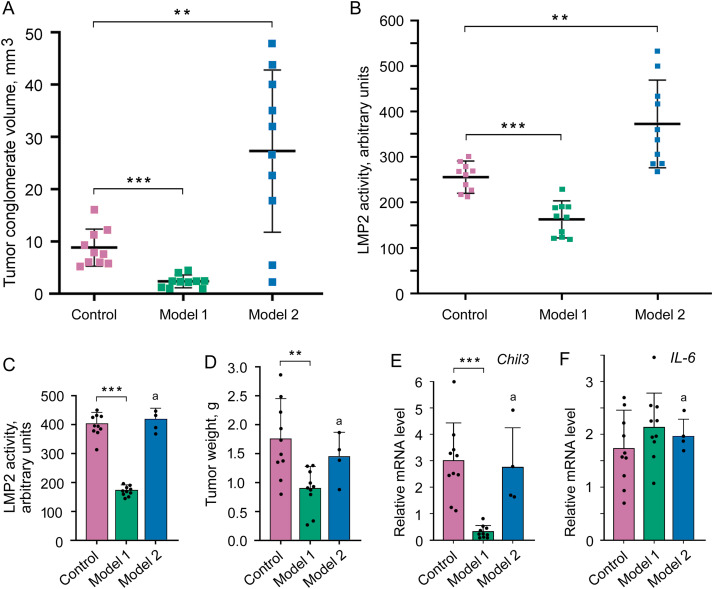
Influence of altered LMP2 activity on the tumor conglomerate formation and development. (**A**) The volume of the tumor conglomerates on day 4 after the transplantation of C26 cells to mice. (**B**) LMP2 activity in tumor conglomerates on day 4. (**C**–**F**) Tumor characteristics on day 21 after the transplantation of C26 cells to mice. (**C**) LMP2 activity in the tumor. (**D**) Tumor weight. (**E**) *Chil3* mRNA expression in the tumor tissue. (**F**) *IL-6* mRNA expression in the tumor tissue. The standard deviation is shown. Significant difference at *p* < 0.01 (**) and *p* < 0.001 (***); n = 10, and n = 4 (a)

To study the role of certain proteins, approaches to complete or partial suppression of their expression/activity are usually used. In the current research, we had a unique opportunity to study the formation of a tumor conglomerate in mouse models under conditions of both suppressed (model 1) and increased LMP2 activity (model 2). In mouse models 1 and 2, as well as in control mice, the tumor conglomerates were formed on day 4 after the subcutaneous transplantation of C26 cells. In model 1, the medium volume of the conglomerates was four times less and in model 2, it was three times larger than in the control (Figs. 5A and S8). We confirmed that LMP2 activity was lower in the conglomerates in model 1 and higher in the conglomerates in model 2 compared to the control (Fig. 5B). This result indicates the significance of the high LMP2 activity of tumor cells for the initial stage of the tumor development—the formation of the tumor conglomerates.

It was possible to investigate the characteristics of the developed tumor in model 1 on day 21 after the transplantation of C26 cells. The activity of the LMP2 subunit was almost 60% lower in the tumor in model 1 than in the tumor of the control group (Fig. 5C), which confirmed the validity of using this model at the indicated period. The tumor weight was two times less in model 1 than in the control group (Fig. 5D). The tumor size was also smaller in model 1 (Fig. S8 and Table S4). Note, the injection of physiological saline solution without KZR-504 into the area of transplanted C26 cells did not influence conglomerate and tumor size on day 4 and day 21 (data not shown).

In addition, we assessed the expression of mRNA of the Chil3 gene, a key marker of immunosuppressive M2 macrophages, and mRNA of the interleukin-6 (IL-6) gene, a pro-inflammatory cytokine [7,8,10] on day 21. Although IL-6 is a marker of M1 macrophages with antitumor function, it can be expressed in a number of other cells within the tumor microenvironment, including tumor-infiltrating immune cells, stromal cells, and tumor cells themselves [22]. In model 1, *Chil3* mRNA expression was strongly (10 times) suppressed in the tumor in comparison with that of the control (Fig. 5E). However, the expression of *IL-6* mRNA did not reliably change (Fig. 5F).

In model 2, six of ten mice with tumors less than 2 cm were sick and refused to eat in the second or third week. Therefore, these animals were euthanized. LMP2 activity in the proteasome pool of the tumor tissue in remaining mice was likely to be restored to the control level by day 21 after C26 cell transplantation (Fig. 5C). In these mice, the tumor weight and size as well as *Chil3* and *IL-6* mRNA expression in the tumor tissue were the same as in the control (Figs. 5D–F and S8, Table S4).

Thus, the results obtained on model 1 indicate the involvement of proteasome subunit LMP2 of the tumor microenvironment (at least M2 macrophages) in the development of the colon 26 adenocarcinoma. At the same time, model 2 allowed us to reveal the participation of proteasome subunit LMP2 localized in C26 cells in the tumor conglomerate formation.

### 3.6 Effect of KZR-504 on Macrophages

**Figure 6:**
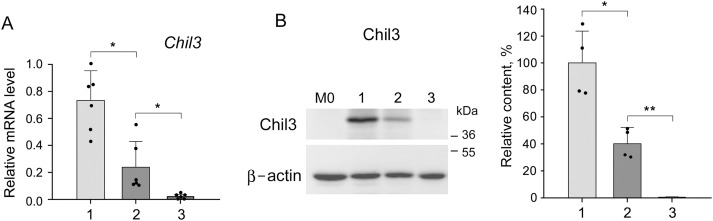

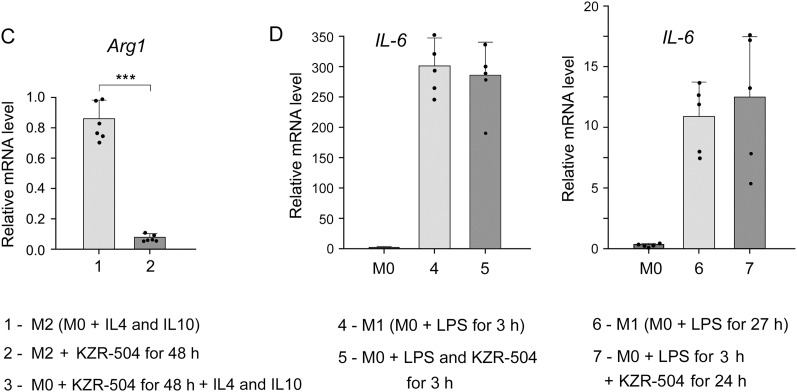
Influence of KZR-504 on the expression of macrophage markers. (**A**) The expression of mRNA of the Chil3 gene. (**B**) The content of Chil3 protein (Western blots and relative amount). The molecular mass of the standard protein markers is shown. The blots were cropped horizontally from gels according to the molecular mass of the proteins studied, and are shown in Fig. S9. (**C**) The expression of the mRNA of the Arg1 gene. (**D**) The expression of mRNA of the IL-6 gene. 1: M2 macrophages, polarized from M0 macrophages, in the culture medium containing a 20 ng/mL cocktail of IL-4 and IL-10. 2: M2 macrophages in the same culture medium + 5 μM KZR-504, incubation for 48 h. 3: M0 macrophages + 5 μM KZR-504, incubation for 48 h, exclusion of KZR-504 (a new portion of the culture medium) + 20 ng/mL cocktail of IL-4 and IL-10, incubation for 24 h. 4: M1 macrophages, polarized from M0 macrophages, in the culture medium containing 100 ng/mL LPS, incubated for 3 h. 5: M0 macrophages + 100 ng/mL LPS and 5 μM KZR-504, incubation for 3 h. 6: M1 macrophages, polarized from M0 macrophages, in the culture medium containing 100 ng/mL LPS, incubated for 27 h. 7: M0 macrophages + 100 ng/mL LPS, incubation for 3 h, + 5 μM KZR-504, incubation for 24 h. The standard deviation is shown. Significant difference at *p* < 0.05 (*), *p* < 0.01 (**), and *p* < 0.001 (***); for (**A**) and (**C**), n = 6, for (**B**), n = 4, and for (**D**), n = 5.

In connection with the results obtained, we were interested in studying the direct effect of LMP2 inhibition on M2-polarized macrophages with the function of immune suppression. M2-polarized macrophages were derived from bone marrow cells of Balb/c mice as described in Materials and Methods. In addition to *Chil3* mRNA expression, we studied the relative content of Chil3 protein. Cultivation of M2 macrophages in the presence of 5 μM KZR-504 resulted in a significant decrease of *Chil3* mRNA expression as well as Chil3 protein content (Figs. 6A,B, S9 and S10, Table S5). This indicates that LMP2 inhibition causes an abnormality of M2 macrophages. Is LMP2 activity necessary only to maintain the functional state of already polarized M2 macrophages, or is it obligatory for the transition of M0 macrophages into M2 macrophages too? To clarify, we cultivated M0 macrophages in the presence of 5 μM KZR-504 for 48 h, excluded KZR-504 by changing the culture medium, and added a 20 ng/mL cocktail of IL-4 and IL-10 for 24 h. In this experiment, *Chil3* mRNA and Chil3 protein were detected in trace amounts (Figs. 6A,B, S9 and S10, Table S5). Therefore, the proteasome LMP2 subunit is a crucial regulator of the expression of Chil3, an M2 macrophage key marker, both at the stage of transition of macrophages from M0 to M2 and at the stage of functioning of polarized M2 macrophages.

Since Chil3 belongs to mouse M2 macrophages [8,23], we drew a parallel with the human immune system by studying the expression of Arg1 gene, a marker of both mouse and human macrophages of the immunosuppressive phenotype [8,24,25]. KZR-504 strongly decreased the expression of the *Arg1* mRNA in polarized M2 macrophages (Fig. 6C).

In addition, it was important to investigate the effect of KZR-504 on M1-polarized macrophages in a culture medium. In these conditions, unlike the tumor microenvironment, IL-6 is a true marker exclusively for M1 macrophages. *IL-6* mRNA expression did not change in the presence of 5 μM KZR-504 (Fig. 6D). Thus, the proteasome LMP2 subunit plays a crucial role in the functioning of M2 macrophages, but not M1 macrophages.

### 3.7 Confirmation of M2 Macrophage Infiltration into Tumor Tissue by Detecting CD163 Cellular Receptor

**Figure 7:**
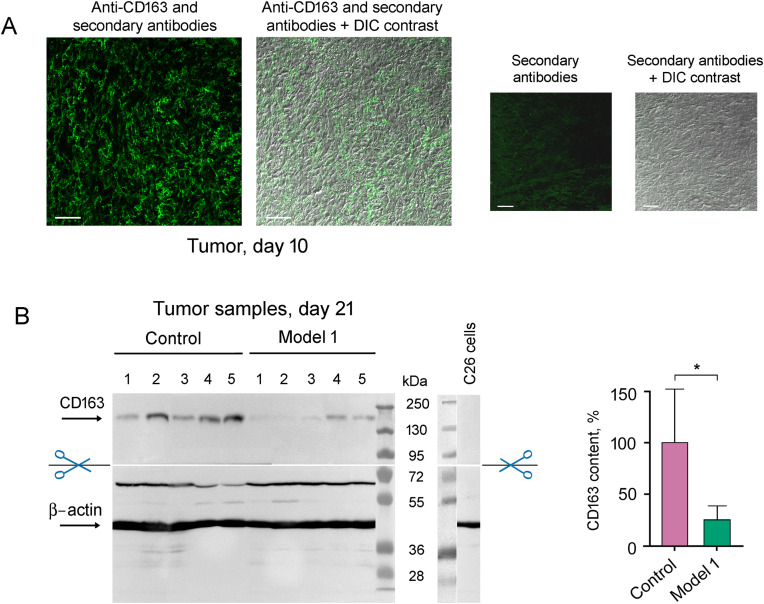
Detection of M2 macrophages infiltrated into tumor tissue with the use of antibodies to CD163. (**A**) Immunohistochemical analysis of the tumor on day 10 after the transplantation of C26 cells to mice. Scale bar, 50 μm. (**B**) Western blot analysis of CD163 content in control tumor samples and tumor samples under 5 μM KZR-504 treatment in model 1 on day 21. The standard deviation is shown. Significant difference at *p* < 0.05 (*); n = 5

As Chil3 is expressed not only by M2 macrophages but also by neutrophils and some other cells [8,23], it was important to confirm M2 macrophage infiltration into tumor tissue by detecting CD163 cellular receptors. Fig. 7A shows the presence of CD163-positive cells in the tumor on day 10 after the transplantation of C26 cells to mice.

CD163 amount is decreased in the tumor after KZR-504 treatment in model 1 (Fig. 7B). Thus, KZR-504 reduces the content of normally functioning M2 macrophages infiltrated in the tumor.

## 4 Discussion

For the first time, we studied the functional significance of proteasome immune subunit LMP2 for the development of gut cancer in mouse models of the colon 26 adenocarcinoma. Our interest in the LMP2 subunit was induced by a high rate of increase in its expression (higher than that of the LMP7 subunit) in patients with rectal cancer [4]. Despite the fact that we have studied a number of features of LMP2 functioning in patients’ rectal adenocarcinoma, mouse models allowed us to use the LMP2 inhibitor to elucidate the role of this subunit *in vivo*.

The proteasomes containing the LMP2 subunit in the tumor cells are very likely to produce special peptides that promote the formation of the tumor conglomerates via activation of matrix metalloproteinase activity or expression. The involvement of the proteasome pool in extracellular matrix regulation is known [26,27]. Our results may indicate a specific participant, the LMP2 subunit, in this process. We plan to study this issue in the future.

The ability of the LMP2 subunit of tumor cells to stimulate the formation of tumor conglomerates from separate cells is important not only for the initial stage of primary tumor development but also for the initial stage of secondary tumor formation during the metastatic process. This indicates that the LMP2 subunit is a promising target for anti-metastatic treatment that may prevent initial metastasis.

**Figure 8:**
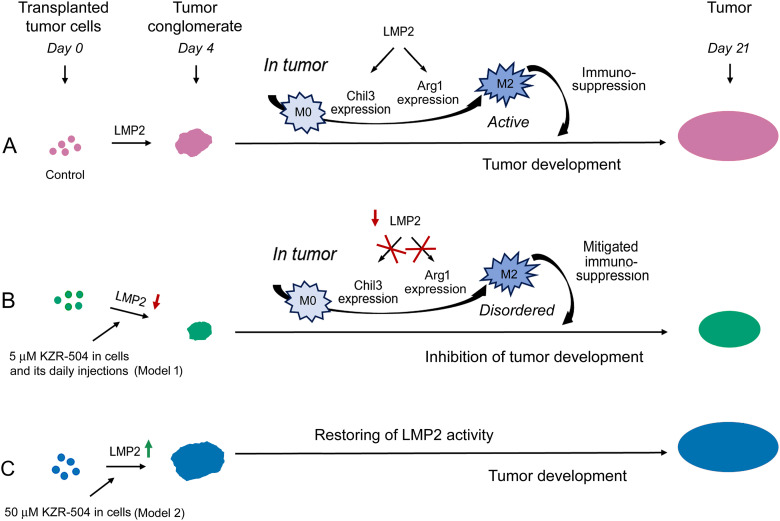
Involvement of proteasome LMP2 subunit in the development of the colon 26 adenocarcinoma in mice. (**A**) Proteasome LMP2 activity in transplanted C26 cells is involved in tumor conglomerate formation. LMP2 activity in cells of the microenvironment is involved in further tumor development. LMP2 subunit stimulates *Chil3* and *Arg1* expression during the polarization of M0 into M2 macrophages and maintains this expression in active M2 macrophages, which in turn inhibit the activity of cytotoxic T lymphocytes. (**B**) Inhibition of the LMP2 activity suppresses tumor conglomerate formation, M0-M2 macrophage polarization, and maintenance of the functional state of already polarized M2 macrophages via blocking the *Chil3* and *Arg1* expression. All these events generally inhibit tumor growth. (**C**) An increase in the activity of the LMP2 subunit in C26 cells stimulates the formation of tumor conglomerates. The restoration of LMP2 activity leads to the formation of tumors, as in the control

Of particular interest are the data on LMP2 involvement in the development of immunological tolerance to tumor cells. The mechanism of ensuring colon adenocarcinoma development in mice, based on our results, is shown in Fig. 8.

As a rule, proteasome LMP2 activity, unlike LMP7 activity, is not considered an independent actor in the immune system. M. Basler and collaborators discovered that the structural property of the LMP2 subunit, rather than its activity, was required for the generation of the male HY-derived CTL-epitope UTY (246–254) [28]. This is true for proteasomes containing LMP7 and LMP2 subunits in a single structure. In the present work, we show for the first time the independent involvement of LMP2 activity in tumor immunity in connection with M2 macrophage functioning.

Tumor macrophages are highly plastic cells that can play either pro- or anti-inflammatory roles in response to environmental signals, including cytokines and growth factors of tumor cells [7]. Macrophages of the M2 type suppress cytotoxic T lymphocyte function by secretion of immunosuppressive factors. Our results show that the macrophage LMP2 subunit stimulates *Chil3* and *Arg1* expression during the polarization of M0 into M2 macrophages and maintains this expression in M2 macrophages (Figs. 5E and 6A–C). Chil3 is a key marker of mouse M2 macrophages [8,23], whereas Arg1 is a marker of both mouse and human M2 macrophages [8,24,25]. This fact allows us to draw a parallel between the results obtained and possible mechanisms of the development of tolerance to tumor cells in patients. Currently, the canonical IL-4/STAT6 pathway of M2 macrophage polarization is well known. *Chil3* and *Arg1* expression are under regulation in this pathway [8,29]. Proteasomes containing the LMP2 subunit are likely to be fine-tuners of the IL-4/STAT6 signaling pathway, but this issue needs to be clarified in detail in the future.

The proteasome LMP2 subunit must not be involved in maintaining the functionality of pro-inflammatory M1 macrophages (Figs. 5F and 6D). This fact makes the LMP2 subunit a suitable target for inactivating the immunosuppressive M2 macrophages in the tumor microenvironment. Thus, we have shown the functional significance of the proteasome LMP2 subunit of both tumor C26 cells and M2 macrophages in the tumor microenvironment for tumor development *in vivo*.

Interestingly, the enhanced activity of the LMP2 subunit was revealed in the proteasome structures containing PA28αβ activator (Fig. 1B). Obviously, the LMP2 subunit, paired with the PA28αβ activator, participates in the regulation of tolerance development. It means that the functioning of the LMP2-PA28αβ proteasome form does not require preliminary ubiquitination of its substrates, which ensures a rapid regulatory process.

The significance of another proteasome immune subunit, LMP7, for alveolar macrophages has been studied earlier by S. Chen and collaborators [30]. Alveolar macrophages from LMP7 knockout mice disclosed a distorted M2 profile upon IL-4 stimulation as characterized by increased M2 marker gene expression and CCL17 cytokine release; however, deficiency of LMP7 did not affect the LPS/IFNγ-triggered M1 profile [30]. In our experiments, inhibition of the LMP2 subunit, on the contrary, caused a serious decrease in the expression of M2 macrophage marker genes. All these facts point to the independent roles of LMP2 and LMP7 subunits in the regulation of M2 macrophage activity.

The strengths of our work are the following. First, we discovered that LMP2 subunit activity was unimportant for intracellular processes, such as mitosis, apoptosis, and viability of colon cancer cells in the culture medium, but was important for intercellular processes in the tumor microenvironment. Second, we realized the functional significance of the proteasome LMP2 subunit for the initial stage of gut cancer development, i.e., formation of tumor conglomerates, and for maintaining the immunological tolerance to tumor cells by stimulating the expression of key markers of immunosuppressive M2 macrophages. Third, we showed that the daily use of the LMP2 inhibitor resulted in a decrease in tumor weight *in vivo*. Fourth, we detected that terminating the LMP2 inhibition led to an increase in its content in cells. This indicates a need for continuous antitumor treatment, targeting the LMP2 subunit until the expected result is achieved.

The major limitation of the work is our not investigating the subtle regulatory mechanisms involving LMP2 activity in the formation of tumor conglomerates.

Our results confirm the importance of a novel research area that increasingly focuses on the tumor microenvironment and new treatment strategies [31]. The data obtained in the current work are the beginning of the development of a new direction, related to the proteasome LMP2 subunit function in the tumor immunity. The work in this field opens prospects for identifying the minute functions of the LMP2 subunit and applying the knowledge gained to medical practice.

## 5 Conclusion

In this work, for the first time, we determined several important facts related to the role of the proteasome LMP2 subunit in the development of gut cancer in mouse models of colon 26 adenocarcinoma. The main findings are the following.

The amount of LMP2 proteasome subunit increases in mouse colon 26 adenocarcinoma tissue compared to the control. This result indicates the LMP2 subunit as an excellent marker of colon adenocarcinoma.

The inhibition of LMP2 activity in colon cancer cells does not influence their viability and proliferation and does not cause apoptosis in the culture medium. However, it suppresses tumor conglomerate formation *in vivo*. On the contrary, the activation of the LMP2 subunit in colon cancer cells stimulates this process.

The LMP2 inhibition suppresses pro-tumor M2 macrophage functioning via the negative influence on the expression of key marker gene *Chil3* in the tumor microenvironment. This leads to the mitigation of immunosuppression and reduces the tumor growth.

The LMP2 inhibition decreases *Chil3* and *Arg1* expression during the polarization of M0 into M2 macrophages and disrupts this expression in M2 macrophages *in cellulo*.

Our current work demonstrates that the LMP2 subunit can be both a reliable marker of colon adenocarcinoma and a target for antitumor treatment. The development of new drugs directed to the irreversible inhibition of LMP2 activity may contribute to suppressing tumor growth. At the same time, our results indicate a need for continuous antitumor treatment with such drugs until the expected result is achieved. In addition, the ability of irreversible LMP2 inhibitors to suppress the formation of tumor conglomerates may be expressed in an anti-metastatic effect.

## Supplementary Materials

Figure S1 Full-size images of gels, fragments of which are shown in Fig. 1A.

Figure S2 All blots for Fig. 1 statistics.

Figure S3 Full-size image of non-denaturing gel, fragment of which is shown in Fig. 1B.

Figure S4 Fluorescence microscopy images, fragments of which are shown in Fig. 3C.

Figure S5 Fluorescence microscopy images, fragments of which are shown in Fig. 3D.

Figure S6 Full-size images of gels, fragments of which are shown in Fig. 4.

Figure S7 All blots for Fig. 4 statistics.

Figure S8 Photos of tumor samples.

Figure S9 Full-size images of gels, fragments of which are shown in Fig. 6.

Figure S10 All blots for Fig. 6 statistics.

## Data Availability

The authors confirm that the data supporting the findings of this study are available within the article and its Supplementary Materials.

## Supplementary Materials

The supplementary material is available online at https://www.techscience.com/doi/10.32604/or.2025.066611/s1.
