# Supplementary figures and images for: Inhibition of Proteasome LMP2 Activity Suppresses Chil3 Expression in Mouse Colon Adenocarcinoma Tissue and Restrains Tumor Growth
Source: Oncol Res. 2025 Aug 28;33(9):2573–95. doi: 10.32604/or.2025.066611 (PMC12408852; doi:10.32604/or.2025.066611)

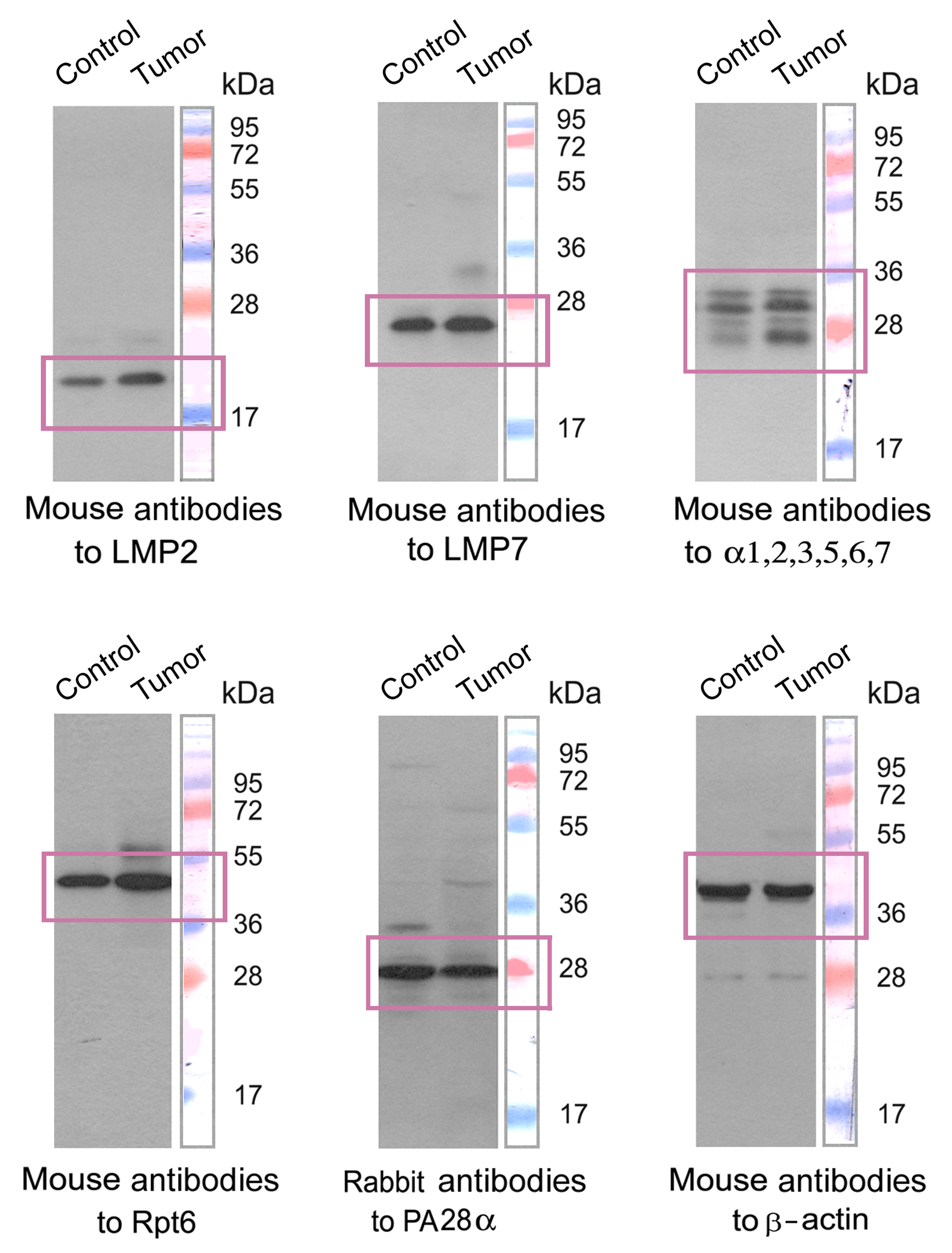

Supplement: Figure S1 [file OncolRes-33-66611-s001.tif]

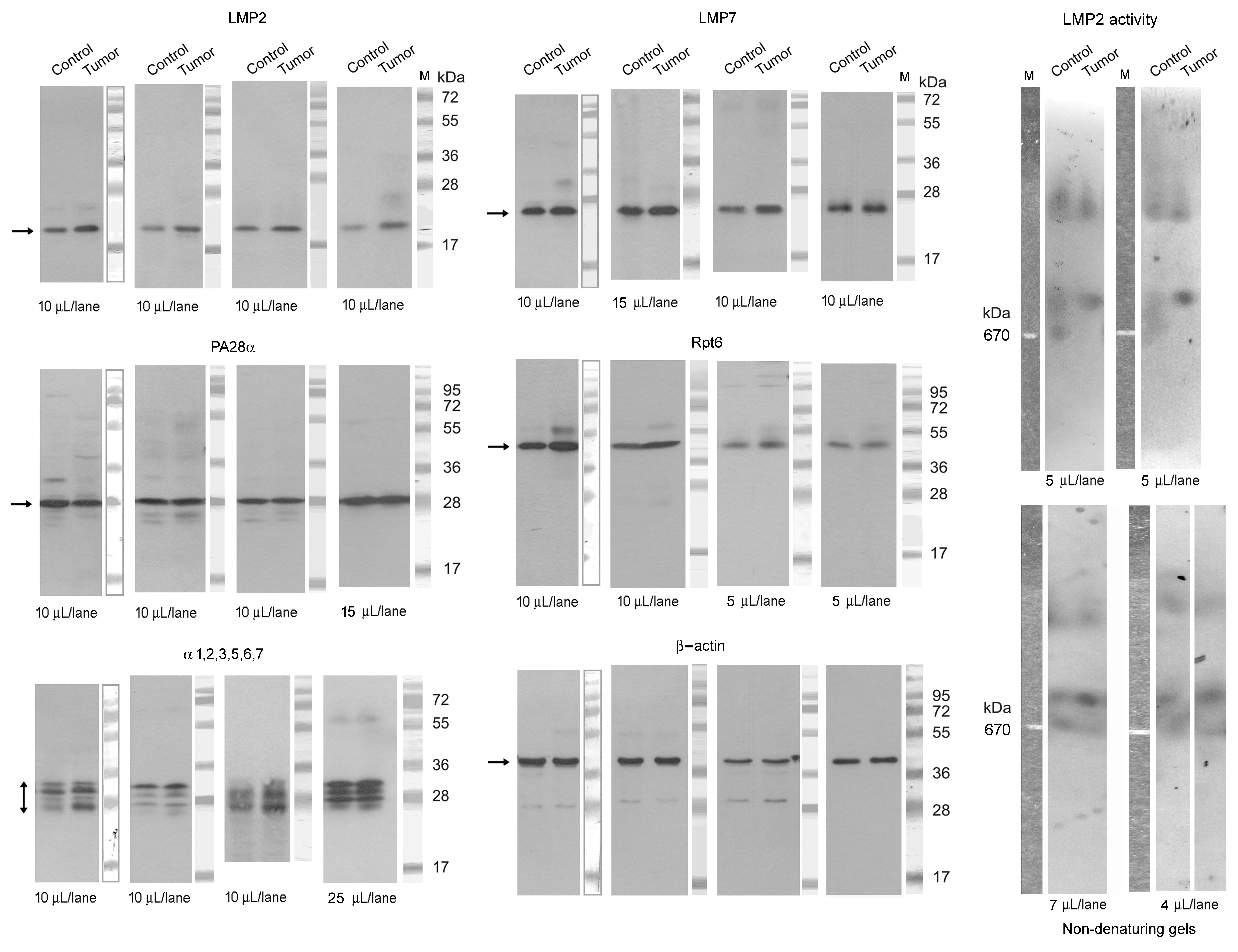

Supplement: Figure S2 [file OncolRes-33-66611-s002.tif]

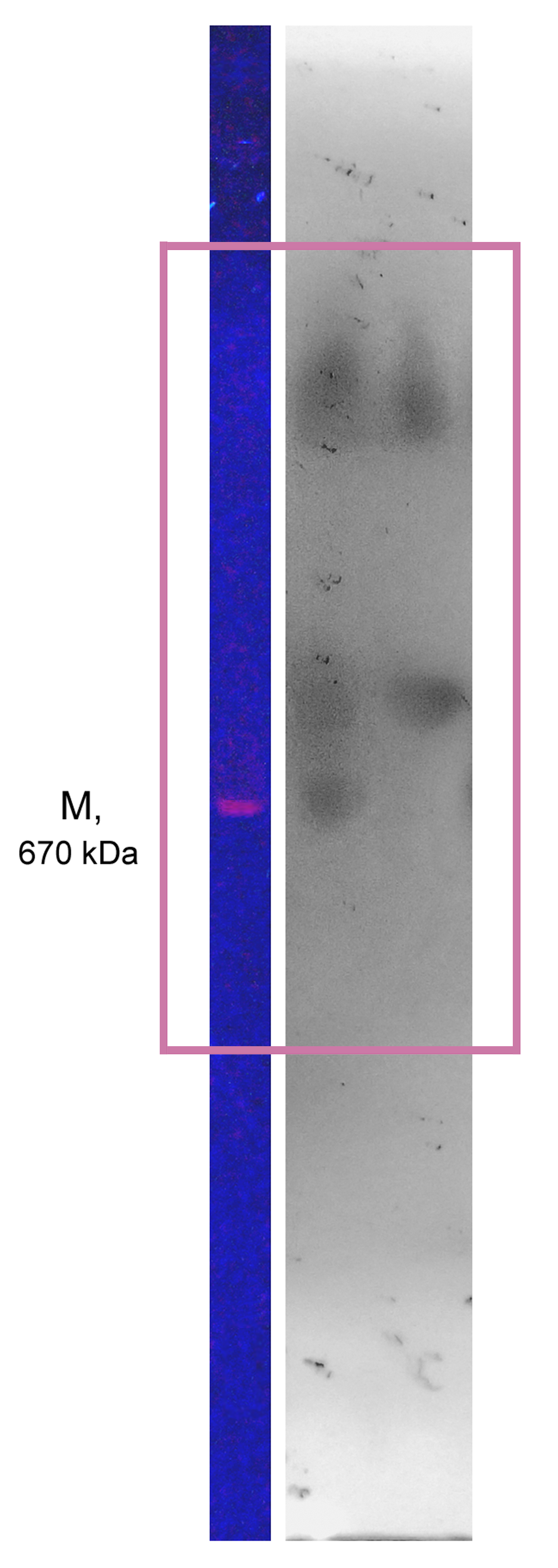

Supplement: Figure S3 [file OncolRes-33-66611-s003.tif]

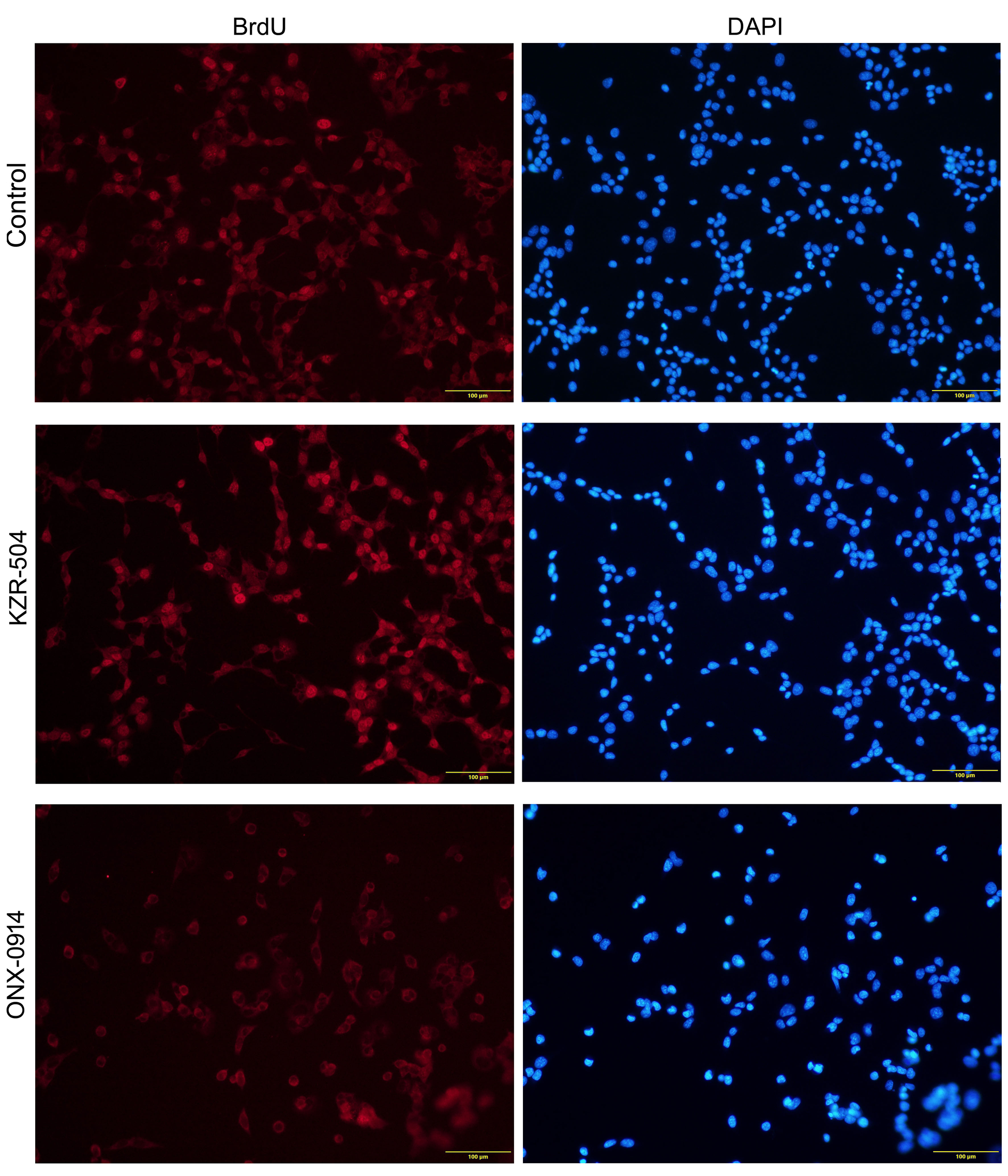

Supplement: Figure S4 [file OncolRes-33-66611-s004.tif]

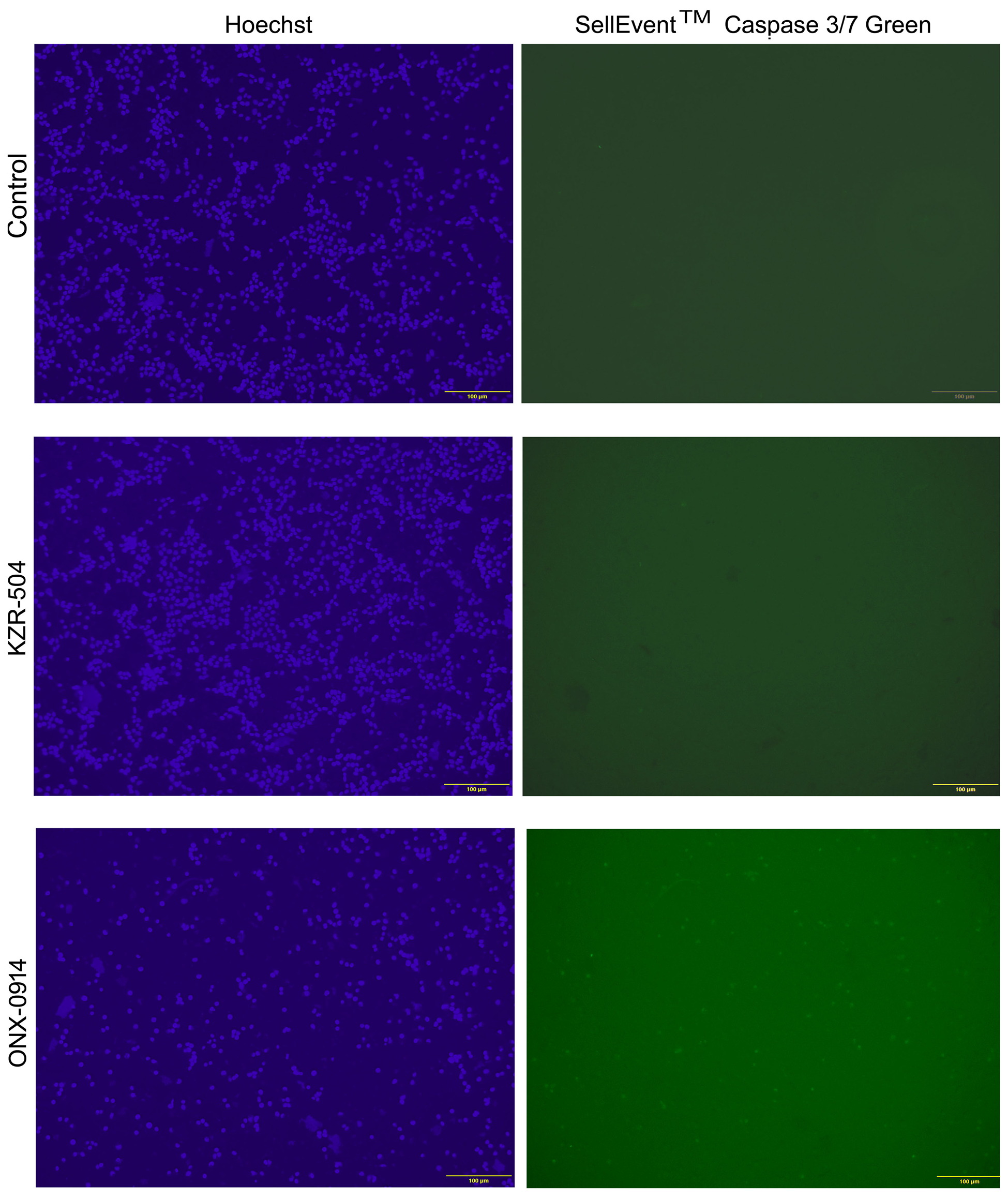

Supplement: Figure S5 [file OncolRes-33-66611-s005.tif]

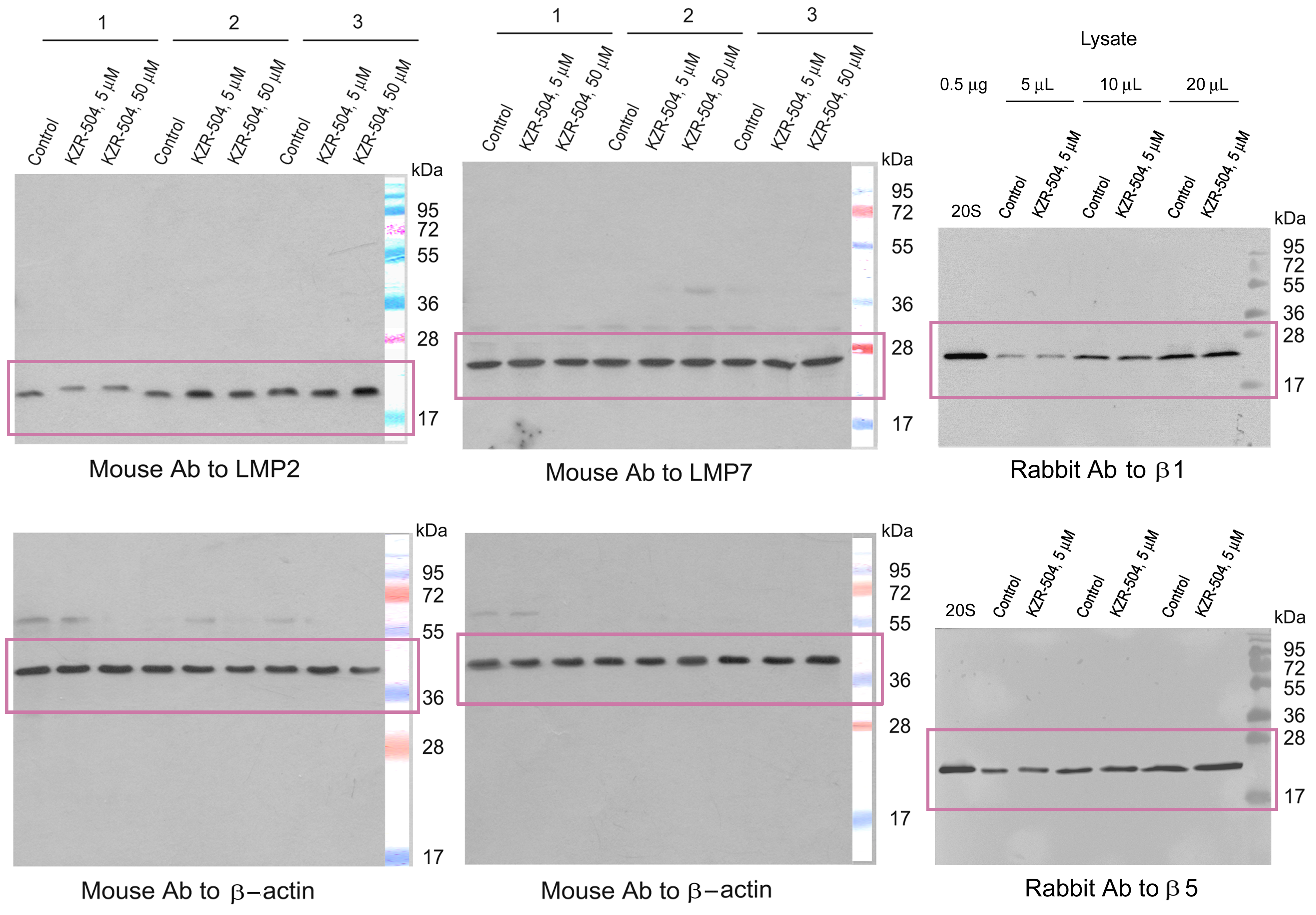

Supplement: Figure S6 [file OncolRes-33-66611-s006.tif]

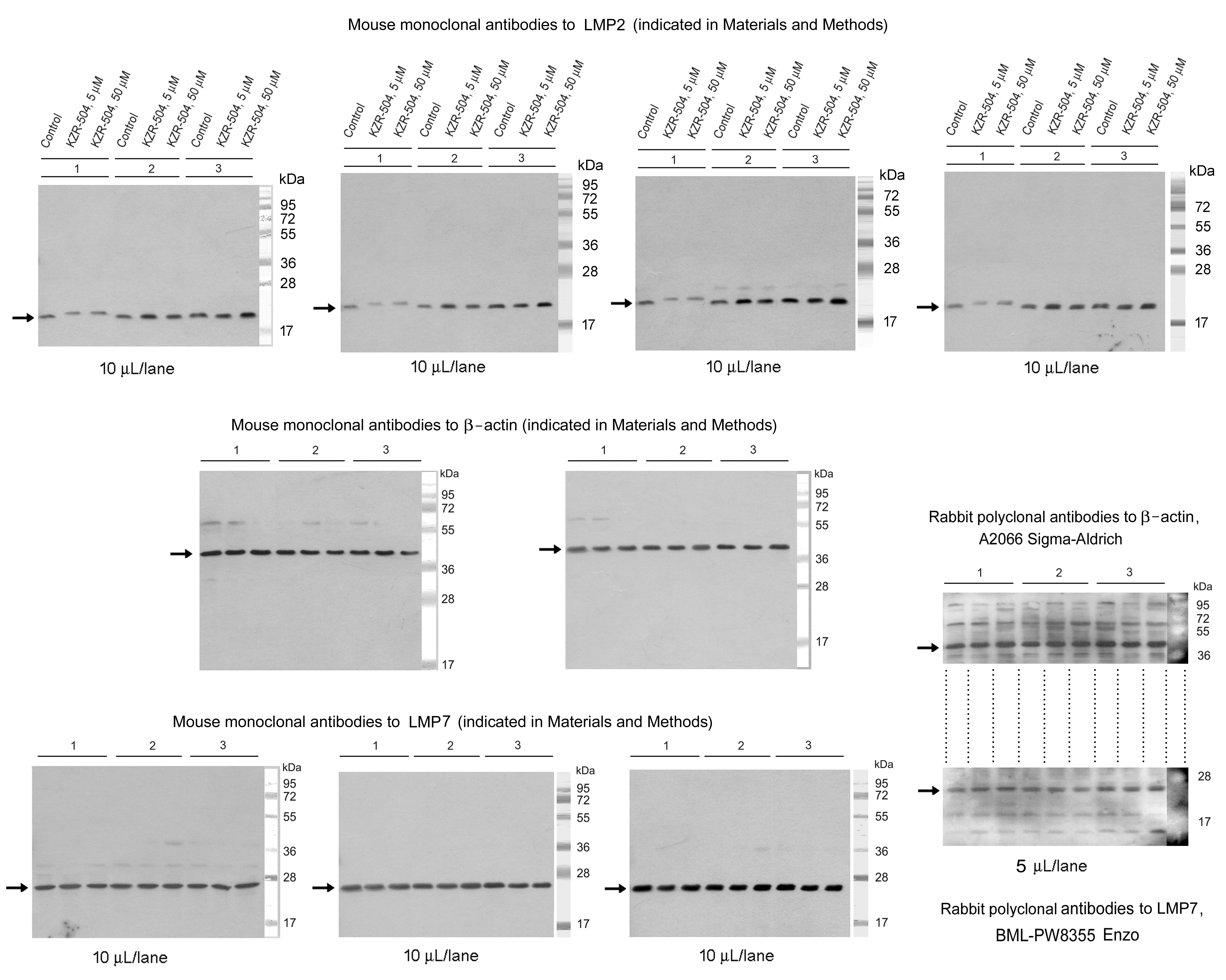

Supplement: Figure S7 [file OncolRes-33-66611-s007.tif]

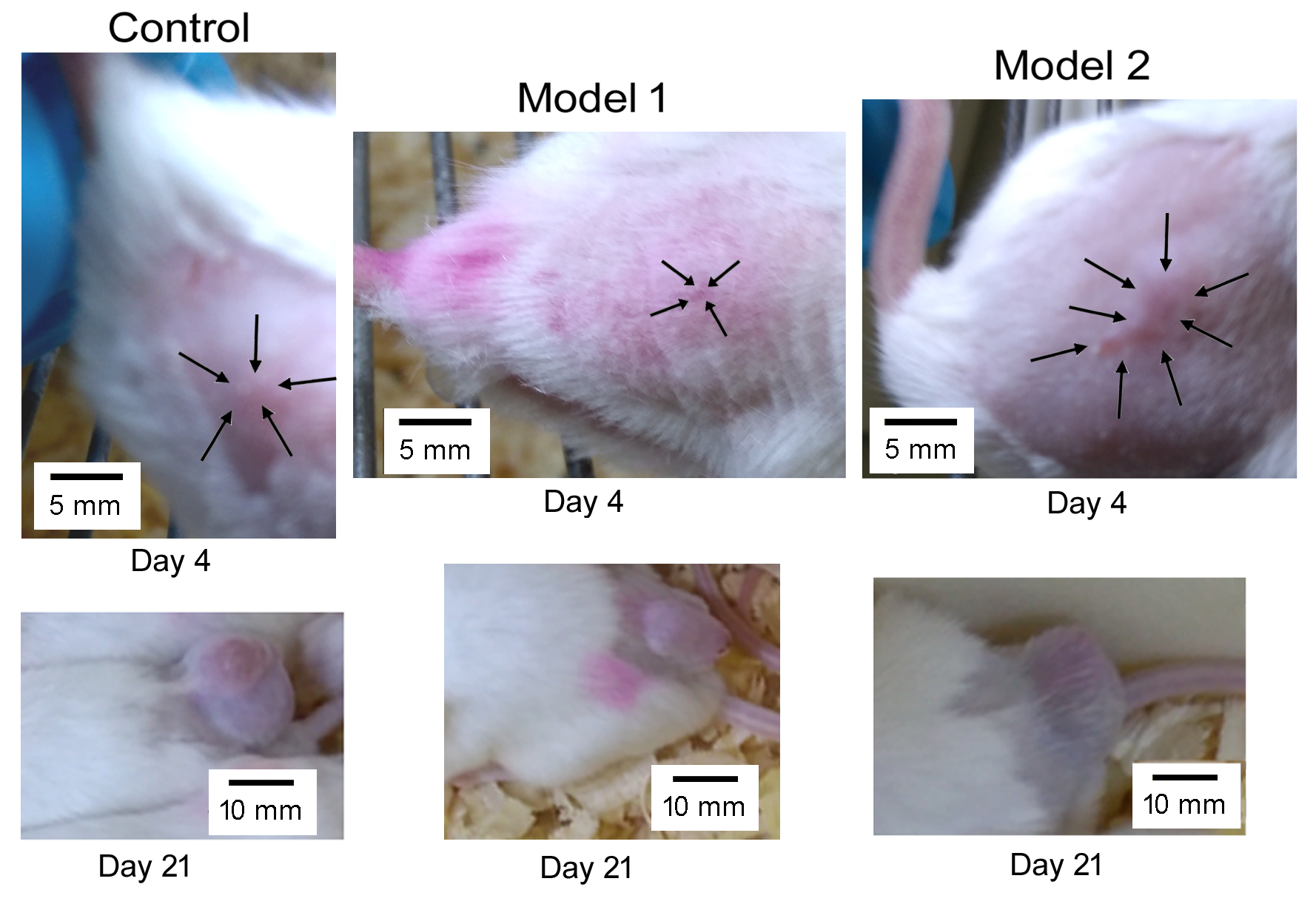

Supplement: Figure S8 [file OncolRes-33-66611-s008.tif]

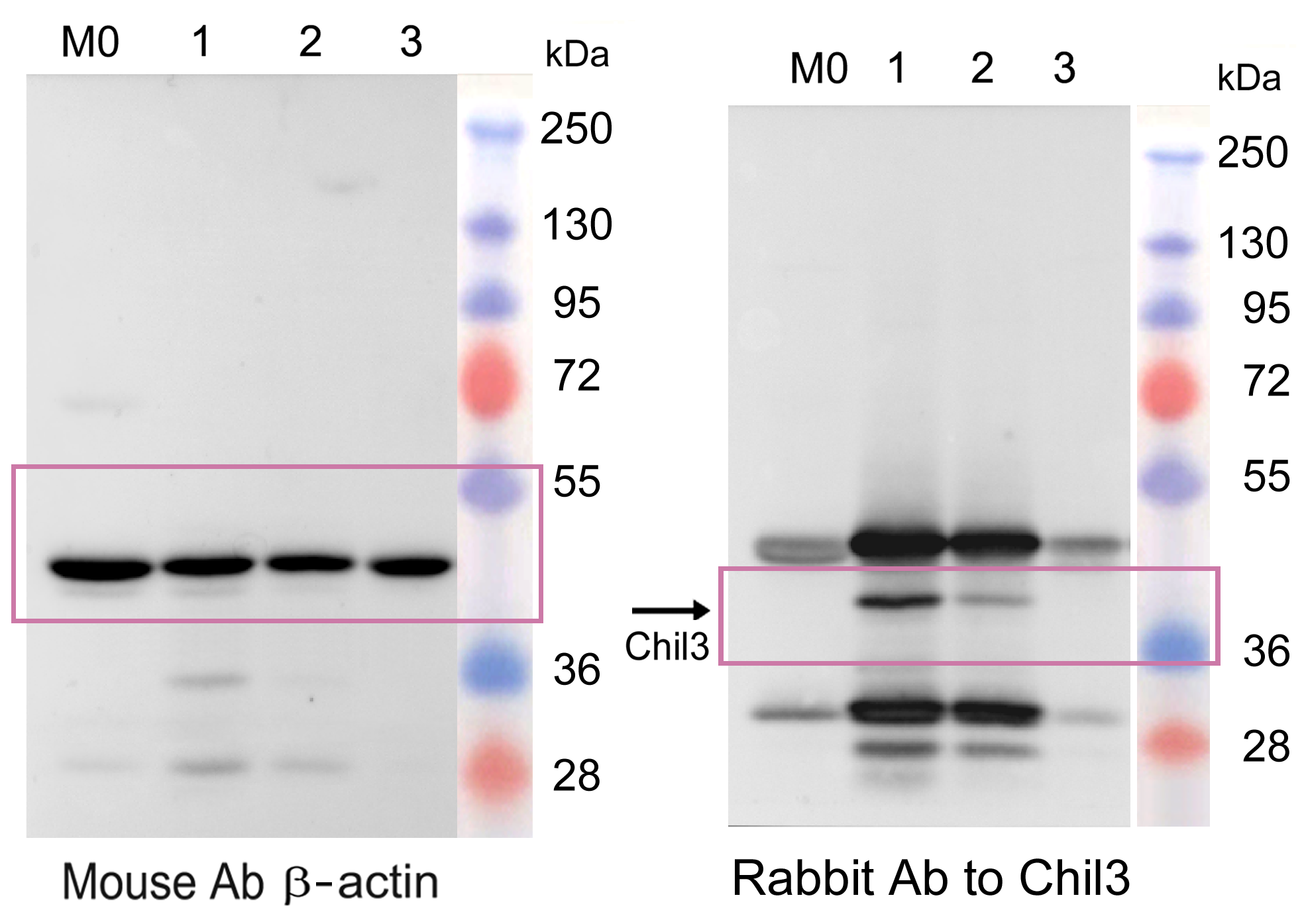

Supplement: Figure S9 [file OncolRes-33-66611-s009.tif]

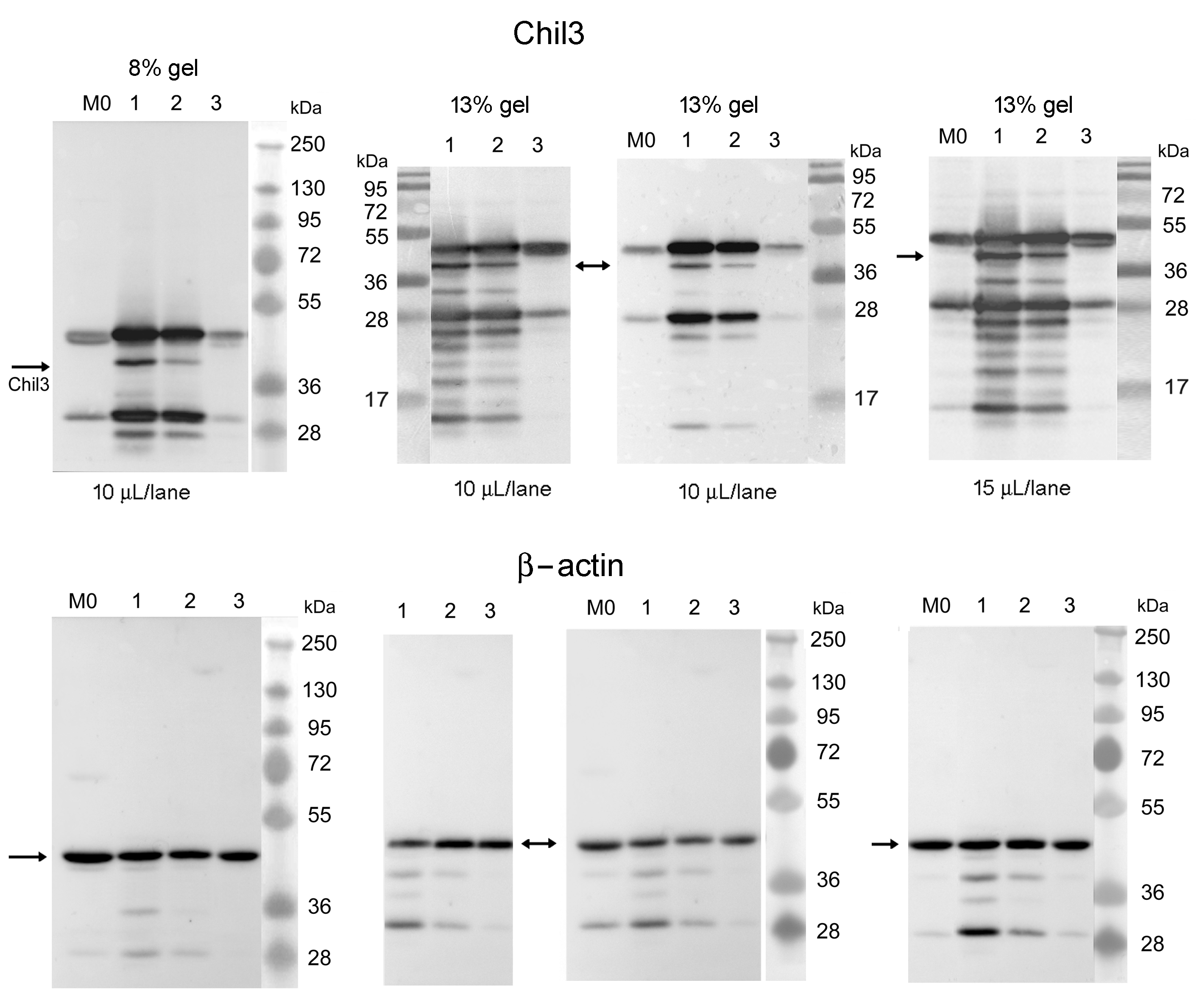

Supplement: Figure S10 [file OncolRes-33-66611-s010.tif]
