# Supplementary material for: Inhibition of Proteasome LMP2 Activity Suppresses Chil3 Expression in Mouse Colon Adenocarcinoma Tissue and Restrains Tumor Growth
Source: Oncol Res. 2025 Aug 28;33(9):2573–95. doi: 10.32604/or.2025.066611 (PMC12408852; doi:10.32604/or.2025.066611)
Supplement: Supplementary file 11 [file OncolRes-33-66611-s011.docx]

**Supplementary+Files:**

**Table S1: Proteasome subunit content in control and tumor tissues on day 21 after transplantation of C26 cells to mice: Blot densities in four experiments.**

| Proteasome subunit | Experiment number | Subunit content | | | |
| --- | --- | --- | --- | --- | --- |
|  |  | Blot densities in arbitrary units per β-actin content in 10 μL of tissue homogenate | | Percent of the average density value | Percent of the control value |
|  |  | Control tissue | Tumor tissue | Control tissue | Tumor tissue |
| α1,2,3,5,6,7 | 1 | 48124 | 67031 | 114 | 139 |
|  | 2 | 33668 | 45745 | 79 | 136 |
|  | 3 | 48791 | 70041 | 115 | 144 |
|  | 4 | 38781 | 40804 | 92 | 105 |
| LMP2 | 1 | 17175 | 29023 | 115 | 169 |
|  | 2 | 13389 | 24219 | 90 | 181 |
|  | 3 | 17107 | 26237 | 114 | 153 |
|  | 4 | 12125 | 24550 | 81 | 202 |
| LMP7 | 1 | 26633 | 34626 | 109 | 130 |
|  | 2 | 21967 | 27113 | 90 | 123 |
|  | 3 | 22746 | 32058 | 93 | 141 |
|  | 4 | 26450 | 28894 | 108 | 109 |
| PA28α | 1 | 26074 | 23951 | 106 | 92 |
|  | 2 | 28229 | 27276 | 114 | 97 |
|  | 3 | 21691 | 20989 | 88 | 97 |
|  | 4 | 22865 | 22397 | 92 | 98 |
| Rpt6 | 1 | 28549 | 35573 | 124 | 125 |
|  | 2 | 22016 | 27880 | 96 | 127 |
|  | 3 | 22704 | 30640 | 99 | 135 |
|  | 4 | 18684 | 21740 | 81 | 116 |

**Table S2: Proteasome LMP2 activity of control and tumor tissues in non-denaturing gel on day 21 after transplantation of C26 cells to mice: Blot densities in four experiments**

| Proteasome fraction in gel | Experiment number | LMP2 activity | | | |
| --- | --- | --- | --- | --- | --- |
|  |  | Blot densities in arbitrary units in 5 μL of tissue homogenate | | Percent of the average density value | Percent of the control value |
|  |  | Control tissue | Tumor tissue | Control tissue | Tumor tissue |
| 20S-19S | 1 | 32049 | 34574 | 119 | 108 |
|  | 2 | 31647 | 29694 | 118 | 94 |
|  | 3 | 22209 | 19184 | 82 | 86 |
|  | 4 | 21866 | 26180 | 81 | 120 |
| 20S-PA28αβ | 1 | 25236 | 56563 | 110 | 224 |
|  | 2 | 25497 | 45106 | 112 | 177 |
|  | 3 | 22650 | 42613 | 99 | 188 |
|  | 4 | 18124 | 35798 | 79 | 198 |
| 20S | 1 | 22293 | 1783 | 80 | 8 |
|  | 2 | 30365 | 3664 | 109 | 12 |
|  | 3 | 26136 | 13151 | 94 | 50 |
|  | 4 | 32509 | 15756 | 117 | 48 |

**Table S3: Effect of KZR-504 on LMP2 and LMP7 content in C26 cells: Blot densities in four experiments**

| Subunit | Experiment number | Content of immune subunits | | | | | | | | | | | | | | | | | | | |  |
| --- | --- | --- | --- | --- | --- | --- | --- | --- | --- | --- | --- | --- | --- | --- | --- | --- | --- | --- | --- | --- | --- | --- |
|  |  | Blot densities in arbitrary units per β-actin content in 10 μL of cell lysate | | | | | | | | | % of average density | % of initial control value | | | | | | | | | |  |
|  |  | Stage 1 (Fig. 4) | | | Stage 2 (Fig. 4) | | | Stage 3 (Fig. 4) | | | Stage 1 (Fig. 4) | | | Stage 2 (Fig. 4) | | | | Stage 3 (Fig. 4) | | | |  |
|  |  | Initial control | KZR-504, 5µM | KZR-504, 50µM | Cont-rol | KZR-504, 5µM | KZR-504, 50µM | Cont-rol | KZR-504, 5µM | KZR-504, 50µM | Initial  control | KZR-504, 5µM | KZR-504, 50µM | | Cont-rol | KZR-504, 5µM | KZR-504, 50µM | | Cont-rol | KZR-504, 5µM | KZR-504, 50µM | |
| LMP2 | 1 | 10267 | 5516 | 7926 | 13041 | 21619 | 21598 | 19668 | 20416 | 28481 | 110 | 51 | 73 | | 120 | 199 | 199 | | 181 | 188 | 262 | |
|  | 2 | 8992 | 3294 | 5166 | 9121 | 18345 | 14510 | 19035 | 17076 | 22565 | 91 | 37 | 57 | | 101 | 204 | 161 | | 212 | 190 | 251 | |
|  | 3 | 9299 | 3261 | 5365 | 11661 | 19411 | 16380 | 19934 | 17568 | 27480 | 94 | 35 | 58 | | 125 | 209 | 176 | | 214 | 189 | 296 | |
|  | 4 | 10491 | 5214 | 9777 | 16071 | 27505 | 20806 | 21945 | 20840 | 29301 | 105 | 50 | 93 | | 153 | 262 | 198 | | 209 | 199 | 279 | |
| LMP7 | 1 | 21090 | 21191 | 20187 | 23690 | 23129 | 21794 | 22362 | 22263 | 23347 | 96 | 101 | 96 | | 112 | 110 | 103 | | 106 | 106 | 111 | |
|  | 2 | 22528 | 21186 | 22912 | 22261 | 22766 | 22412 | 24422 | 23083 | 23267 | 103 | 94 | 102 | | 99 | 101 | 100 | | 108 | 103 | 103 | |
|  | 3 | 25322 | 24128 | 26764 | 26701 | 25294 | 27738 | 30419 | 28565 | 26903 | 116 | 95 | 106 | | 105 | 100 | 110 | | 120 | 113 | 106 | |
|  | 4 | 18540 | 17492 | 20290 | 19126 | 19626 | 19302 | 24744 | 23998 | 27582 | 85 | 94 | 109 | | 103 | 106 | 104 | | 133 | 129 | 149 | |

**Table S4: Dimensions of tumors in mice on day 21 after transplantation of C26 cells**

| Mouse | Tumor dimensions, mm × mm × mm | | |
| --- | --- | --- | --- |
|  | Control | Model 1 | Model 2 |
| 1 | 17 × 14 × 8 | 15 × 14 × 6 | 17 × 14 × 10 |
| 2 | 15 × 15 × 13 | 14 × 12 × 8 | 16 × 16 × 12 |
| 3 | 19 × 18 × 10 | 12 × 10 × 7 | 13 × 10 × 6 |
| 4 | 13 × 10 × 8 | 10 × 7 × 4 | 20 × 16 × 12 |
| 5 | 14 × 10 × 8 | 13 × 13 × 8 |  |
| 6 | 20 × 14 × 12 | 12 × 10 × 6 |  |
| 7 | 18 × 14 × 10 | 10 × 10 × 8 |  |
| 8 | 17 × 15 × 7 | 13 × 10 × 7 |  |
| 9 | 19 × 19 × 10 | 10 × 6 × 4 |  |
| 10 | 18 × 14 × 12 | 10 × 10 × 6 |  |

**Table S5: Influence of KZR-504 on Chil3 content in macrophages: Blot densities in four experiments**

| Experiment number | Chil3 content | | | | | |
| --- | --- | --- | --- | --- | --- | --- |
|  | Blot densities in arbitrary units per β-actin content in 10 μL of cell lysate | | | Percent of the average density value | Percent of the control value | |
|  | M2 (M0 + IL4 and IL10)  (Control) | M2  + KZR-504 | M0  + KZR-504 + IL4 and IL10 | M2 (M0 + IL4 and IL10)  (Control) | M2  + KZR-504 | M0  + KZR-504 + IL4 and IL10 |
| 1 | 4346 | 1350 | 42 | 129 | 31 | 0.97 |
| 2 | 2720 | 1451 | 24 | 81 | 53 | 0.89 |
| 3 | 2674 | 860 | 19 | 79 | 32 | 0.72 |
| 4 | 3747 | 1866 | 14 | 111 | 50 | 0.38 |


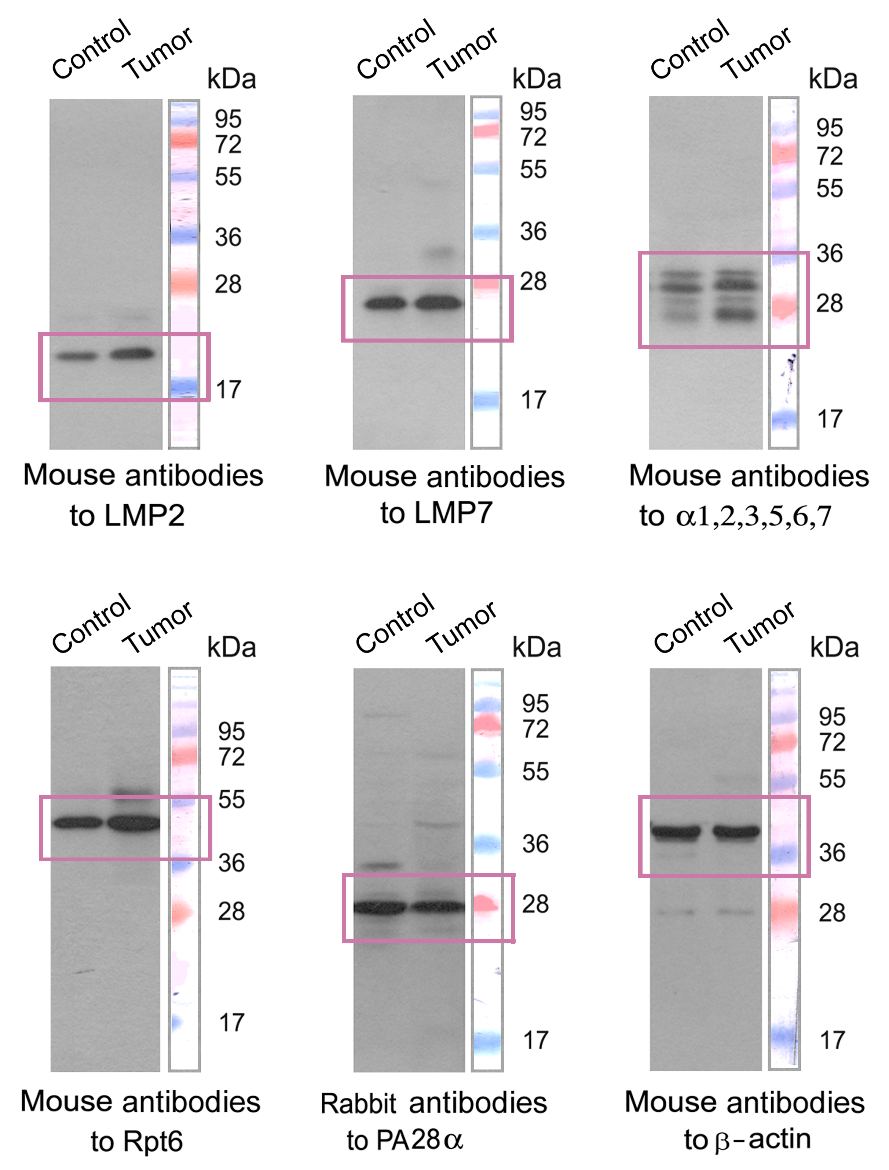


**Figure S1:** Full-size images of gels, fragments of which are shown in Fig. 1A.





**Figure S2:** All blots for Fig. 1 statistics.


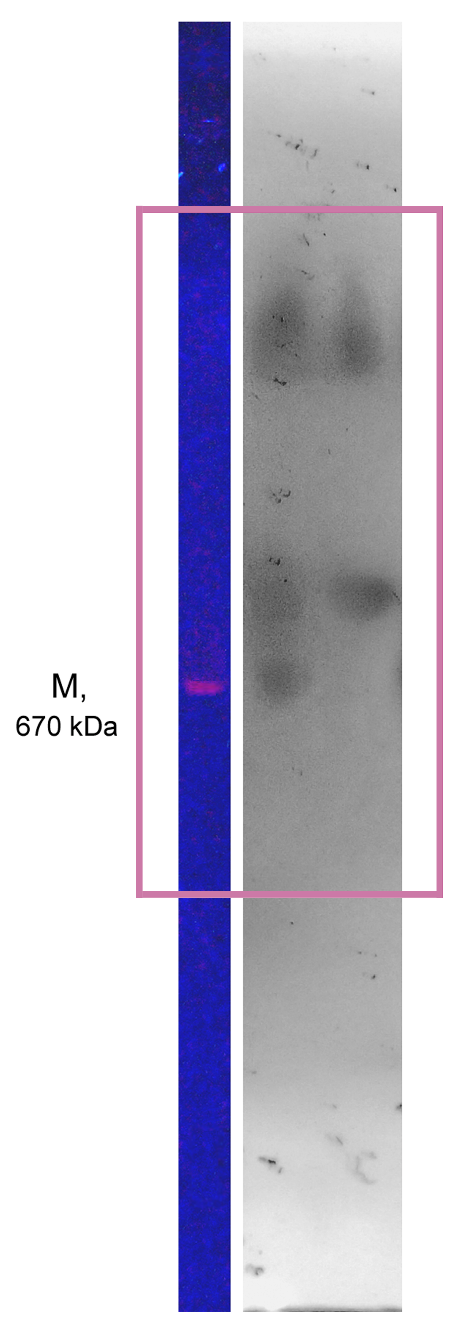


**Figure S3:** Full-size image of non-denaturing gel, fragment of which is shown in Fig. 1B.


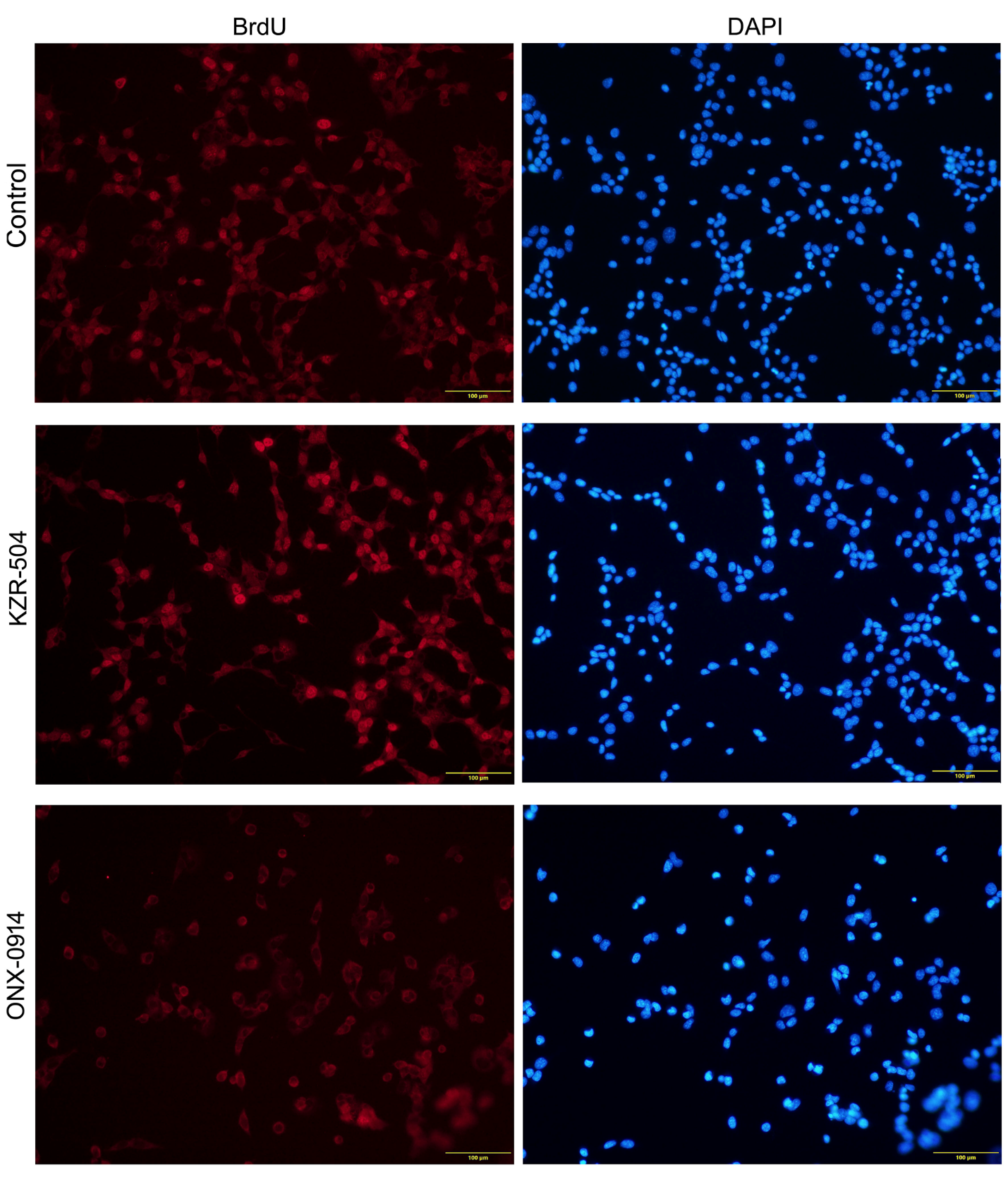


**Figure S4:** Fluorescence microscopy images, fragments of which are shown in Fig. 3C.


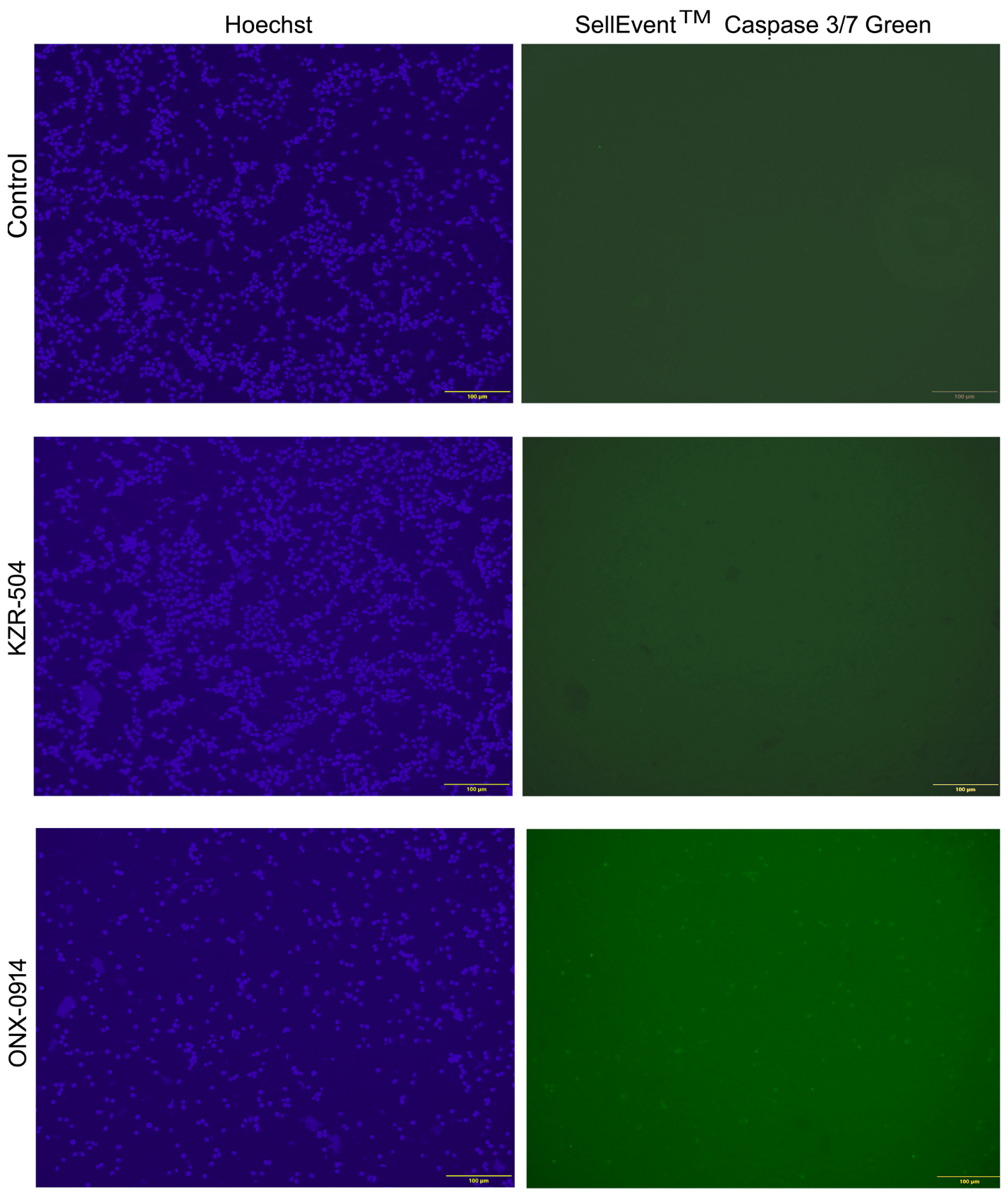


**Figure S5:** Fluorescence microscopy images, fragments of which are shown in Fig. 3D.


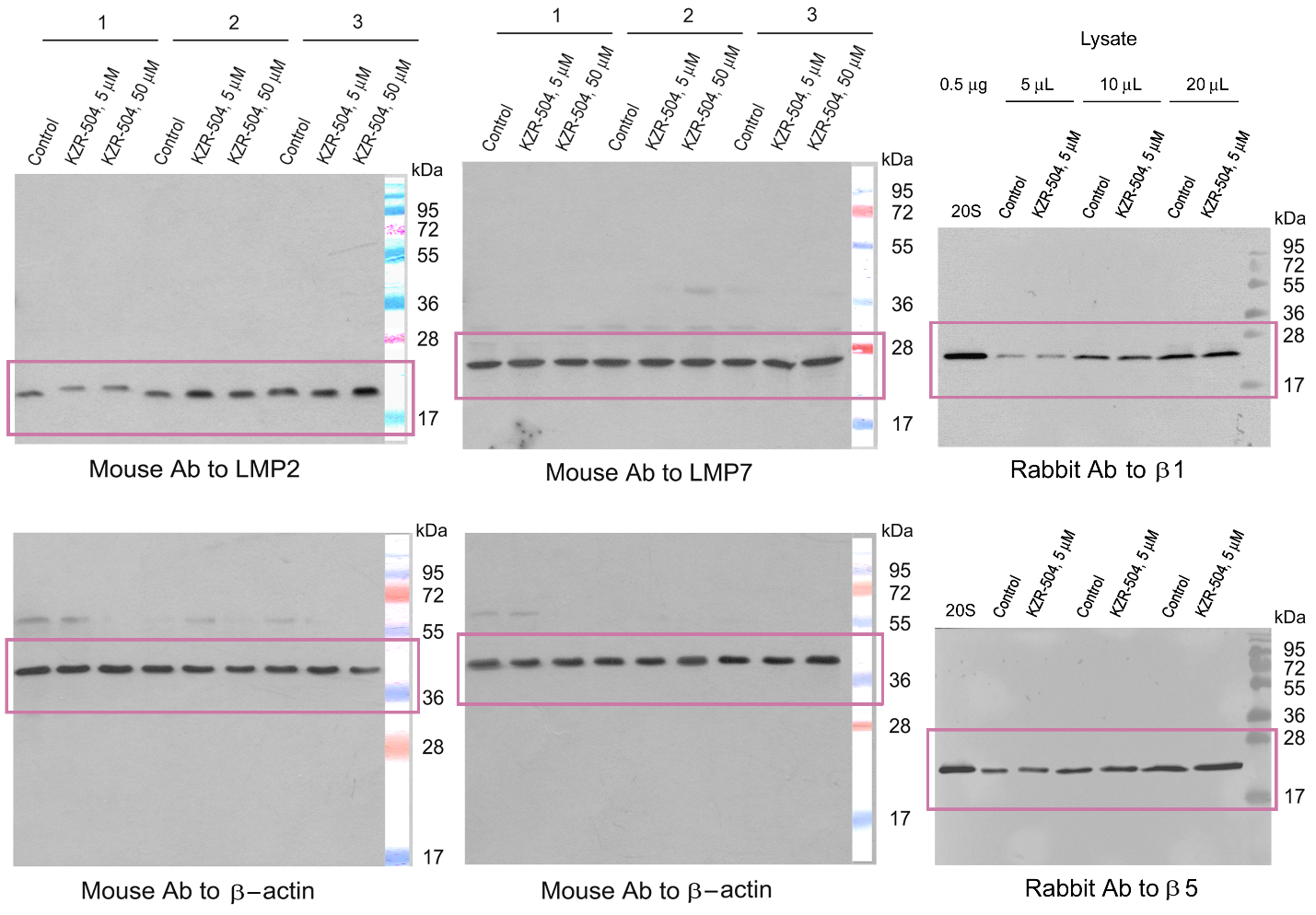


**Figure S6:** Full-size images of gels, fragments of which are shown in Fig. 4.





**Figure S7:** All blots for Fig. 4 statistics.


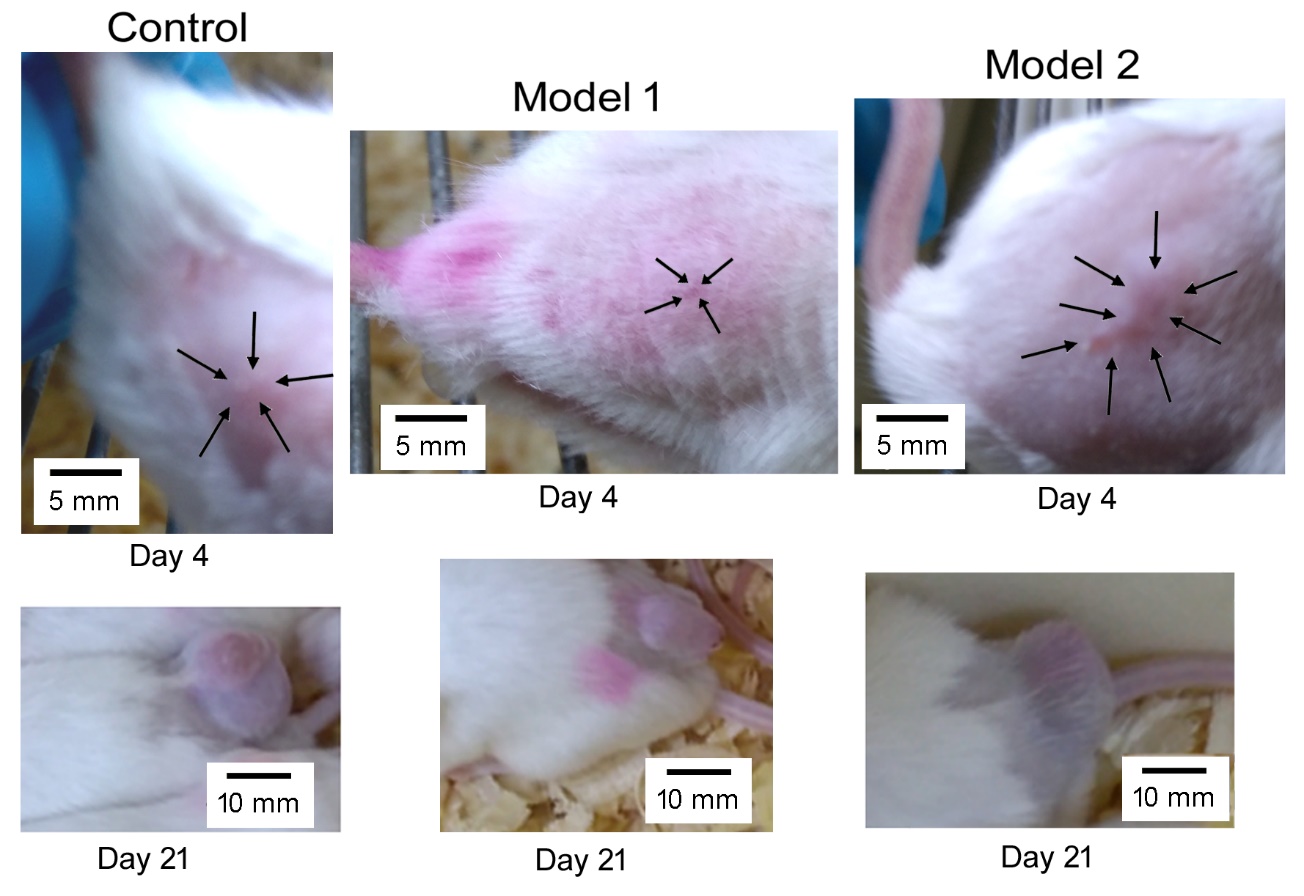


**Figure S8:** Photos of tumor samples.


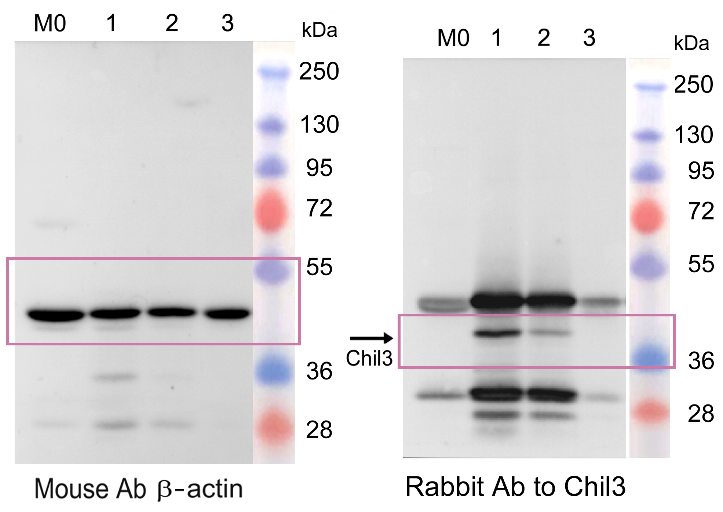


**Figure S9:** Full-size images of gels, fragments of which are shown in Fig. 6.





**Figure S10:** All blots for Fig. 6 statistics.
